# Exploration of fathers’ mental health and well-being concerns during the transition to fatherhood, and paternal perinatal support: scoping review

**DOI:** 10.1136/bmjopen-2023-078386

**Published:** 2024-11-12

**Authors:** Ashleigh Elizabeth Watkins, Catherine El Zerbi, Ruth McGovern, Judith Rankin

**Affiliations:** 1 Population Health Sciences, Newcastle University, Newcastle upon Tyne, UK; 2 Population Health Sciences Institute, Newcastle University, Newcastle upon Tyne, UK

**Keywords:** Postpartum Period, Anxiety disorders, PUBLIC HEALTH, QUALITATIVE RESEARCH

## Abstract

**ABSTRACT:**

**Objectives:**

To appraise and synthesise qualitative literature identifying: (a) fathers’ concerns and challenges during the transition to fatherhood that may be impacting mental health and well-being experiences; and (b) fathers’ experiences of antenatal programmes, and psychological and social support, to understand how we can better support fathers in addressing these concerns.

**Design:**

International, qualitative evidence synthesis, scoping review.

**Data sources:**

Six electronic databases (Medline, Embase, PsycINFO, CINAHL, Scopus, ASSIA) alongside 'grey' and supplementary searches were conducted March 2024.

**Eligibility criteria:**

Qualitative studies and qualitative data extracted from mixed methods studies focusing on fathers’ mental health within the perinatal period globally were included.

**Data extraction and synthesis:**

The recommended Joanna Briggs Institute data extraction and critical appraisal tools were used, and an inductive thematic synthesis approach employed.

**Results:**

37 qualitative studies were included: UK (n=11), Europe (n=9), Australia (n=7) and Asia (n=6), USA (n=1), Canada (n=1) and the Middle East (n=2). Quality appraisal scores were moderate to high (5–10). Four analytical themes and eight subthemes were generated: (1) diminished partner relationship, (2) provider or protector? Multiplicity of fatherhood identification, (3) forgotten entity within the perinatal experience and (4) "I try to battle it myself," masculine ideals within fatherhood. The findings highlighted that fathers found the transition to be difficult, compounded by insecurity within their role as both partner and father. Fathers found a paucity of paternally focused support and antenatal programmes, not addressing fathers’ needs within the transition.

**Conclusions:**

The many concerns and challenges highlighted in the review demonstrate the importance of understanding the negative impact of the transition on fathers’ mental health and well-being. There is a need for greater attention to fathers’ experiences of paternal perinatal support and programmes through research and practise to inform future interventional development.

**Trial registration:**

PROSPERO: CRD4202231381.

**STRENGTHS AND LIMITATIONS OF THIS STUDY**

- This review included qualitative studies with varied qualitative methodologies, including methods of data collection, publication date, population or language, and comprehensive search of all fathers from different backgrounds.
- Active involvement of a patient and public involvement group of four fathers was incorporated within the analytical stages of the review, and contributed to the overall validity of the conclusions and interpretations of the findings from a public perspective.
- Studies were not removed because of poor quality but reported in accordance with the Joanna Briggs Institute criteria; studies were still included that may not have presented as valid findings as others due to the exclusion of qualitative requirements.
- Most of the included studies were from westernised cultures (n=29), limiting the review findings to this population, as well as restricting global perspectives and cultural comparisons (n=8) across studies.

## Background

 Research has established a relationship between pregnancy and childbirth and elevated depressive and anxiety symptoms, with fathers more likely to have depression and anxiety than the average for men.^1^,^3^ The transition to fatherhood (defined as physical, psychological and social changes in the lives of fathers from as early as conception to after birth) can be a stressful and isolating experience, demanding significant lifestyle changes that can have considerable negative impacts on fathers’ mental health and well-being.^4^ Paternal perinatal mental health refers to the occurrence of mental health conditions, such as postnatal depression and anxiety, in fathers in the perinatal period (pregnancy and the first 12 months after childbirth).^5^ One in 10 fathers experience postnatal depression.^6^

It is postulated that many lifestyle adjustments within the transition to fatherhood and increased demands on fathers’ resources during the perinatal period has possibly increased their vulnerability to developing mental health conditions.^7^ A systematic review of 32 studies exploring fathers’ psychological transition to fatherhood, states pregnancy and childbirth as being a psychologically impactful period due to the reorganisation of self, and the rollercoaster of emotions experienced up until birth.^8^ Further research has also identified the postnatal period (1 year after birth) as potentially being the most interpersonally challenging period, due to balancing both work–life and home–life responsibilities and concerns, including societal and economic pressures.^4 8 9^

Evidence highlights the necessity for antenatal programmes and support tailored to fathers.^10^ Throughout qualitative research, fathers have recalled concerns regarding the absence of adequate support and poor information.^11^ Past research exploring antenatal birth education programmes have resulted in decreases in anxiety and psychological distress within the postnatal period from a quantitative stance.^12 13^ However, the limited qualitative research that has explored fathers’ experiences of support and education programmes have stated that support is not currently addressing fathers’ specific needs.^4 14^ This emphasises the need for further research to explore these perspectives to better support design and delivery of paternal perinatal support.

While past research has been conducted, paternal perinatal mental health and well-being are still under-researched, despite the importance of fathers’ contribution in the perinatal period. Further research is needed to address gaps that remain.^15^ The most recent systematic review was conducted 6 years ago with other limited research up to 15 years ago.^8^,^10^ It is imperative that, due to our understanding of operationalisation of fatherhood continuing to change within society, research is up to date to eliminate possible recent findings excluded within systematic reviews. Past systematic reviews have included studies in western societies alone (referring to the adoption of practises, behaviour and culture of Western Europe/North America by societies and countries in other parts of the world), identifying a non-homogeneous focus in all western societies, with many of the included studies conducted within limited countries, or have highlighted that studies included within reviews lacked ethnic and cultural diversity.^8^,^1016^ It is plausible that because certain reviews excluded non-English studies, fathers’ mental health and well-being experiences in the perinatal period could not be elicited from an international perspective, restricting common patterns of data interpretations due to cultural differences.^8 9^ Inclusion of all studies (non-English) in future systematic reviews is recommended for further intuition.^8^ The previous literature has also advised further exploration of experienced fathers’ mental health viewpoints, as well as first time fathers.^8 9^

Previous systematic reviews exploring fathers’ mental health experiences in the perinatal period did not explicitly capture fathers’ experiences of involvement in specific paternal perinatal mental health interventions or programmes as types of catered support; reviews that have additionally captured this have adopted quantitative approaches assessing depression symptomology rather than subjective experiences.^8^,^10^ Previous evidence advises exploring qualitatively what types of support fathers want and what interventions would be acceptable.^8^ To date, there are no National Institute for Health and Care Excellence (NICE) guidelines on interventional support specifically for paternal perinatal mental health. This is of concern considering the growing problem of fathers’ poor mental health within this period.

Firstly, this review will focus on questions including, how do fathers experience the transition to fatherhood mentally and what are fathers’ experiences of current paternal perinatal support? To address the above gaps, this review will specifically explore the updated international literature, identifying fathers of differing backgrounds (first time and experienced), and the concerns and challenges within the transition to fatherhood that may be impacting mental health and well-being (emotional and/or social) experiences. Secondly, this review will also explore fathers’ subjective experiences of accessing antenatal programmes and psychological/social support and interventions, to capture their perspectives on what aspects of support are acceptable or not currently addressing fathers’ support needs.

## Methods

This qualitative evidence scoping review was designed to identify international literature relating to fathers’ mental health and well-being within the transition to fatherhood and paternal perinatal support, adopting a thematic synthesis approach.^20^ A scoping review was chosen due to the exploration of multiple questions and broad scope in research aim, providing an overview and map of the evidence and allowing for identification of future research initiatives.^21^ Scoping reviews use the same degree of rigour as systematic reviews, following the Preferred Reporting Items for Systematic reviews and Meta-Analyses extension for Scoping Reviews (PRISMA-ScR) checklist. A protocol was developed and registered with the international prospective register of systematic reviews (PROSPERO). The second objective has been modified from the original protocol objective to suit a qualitative scoping review inclusion, as the objective indicated the possible requirement of quantitative studies.

### Search strategy

The search strategy aimed to identify published and unpublished studies that met the required eligibility criteria; table 1 outlines the review’s inclusion and exclusion criteria for selection of studies. Relevant qualitative studies were identified through a three step search strategy. Limited/primary searches with Google Scholar were conducted first to identify keywords in the titles, abstract and index terms of studies relevant to the review question. A comprehensive search of six databases (Medline, Embase, PsycINFO, CINAHL, Scopus and ASSIA) using all identified keywords was then conducted. The search strategy incorporated both subject headings (MESH terms) and the developed keywords ('fathers' OR 'paternal') identified within the titles and abstracts alone (see online additional file 1 for the full search strategy). Development of the search strategy was informed by an information scientist at Newcastle University library. This was tested against tracer papers expected to be found within the search results to ensure confidence in the search strategy. No restrictions on years or language were used. A manual search on Google was also conducted to identify any 'grey' literature not captured within peer reviewed databases (OATD and relevant websites/charities). Thirdly, reference lists of all identified studies were searched for additional studies, and final citation searches were conducted. Searches were conducted from inception to August 2022, with a final re-run of searches from inception to March 2024.

**Table 1:** Inclusion and exclusion criteria

	Inclusion	Exclusion
Participants/population	Fathers (an individual who has parental responsibility of a child; this can include biological, adoptive fathers or stepfathers) in the perinatal period (defined as conception to 12 months postpartum)	Did not directly relate to fathers’ well-being/mental health within the perinatal period
Interventions/exposure	Qualitative studies that considered new or recent fathers’ mental health/well-being concerns within the transition to fatherhood (any fatherhood concerns/challenges impacting fathers overall mental health/well-being, referring to emotional and/or social well-being) or explored fathers experiences of current antenatal programmes/support in addressing these fatherhood mental health/well-being concerns in the perinatal period	Studies of men with severe mental illness before becoming fathers within the perinatal period or that focused on experiences of specific events in the perinatal period, such as perinatal loss, birth trauma, neonatal intensive care experiences and COVID-19

### Eligibility criteria

Studies were included if they met the criteria described in table 1.

### Selection and data extraction

The database searches were conducted by the first author (AEW). Studies were imported into Endnote V.X20 and de-duplicated, then imported into the Rayyan online programme for systematic/scoping reviews, and de-duplicated a final time. Studies were independently screened by the first author (AEW) against the eligibility criteria. A second reviewer (AK, fellow PhD student) screened (10%) the titles and abstracts of the studies independently; after two online meetings (with AEW), going through each abstract, agreed irrelevant studies were removed. The second reviewer then screened a further 10% of the full text articles independently. Any discrepancies between the two reviewers were discussed in an additional meeting and included studies were agreed upon. The meetings involved each reviewer justifying their case and re-reading the article together until consensus was reached.

Qualitative data were extracted (by AEW) from included studies using a standardised data extraction table based on the Joanna Briggs Institute (JBI) data extraction tool, modified to respond to the review question.^22^ The data extraction table was designed and piloted by AEW and further piloted by the second reviewer (10%) before full data extraction. The data extracted included: details on methodology, methods, phenomena of interest/aim, sample size/participants characteristics, setting/country and data analysis.

Data were extracted from the data sources findings' sections of all included studies for the thematic synthesis. This included quotations from study participants reported in published papers, aggregated survey/questionnaire data and authors interpretations, inclusions and implications. Types of data were extracted due to relevance to the review objectives and significance to contributing to the qualitative evidence synthesis. The research team (AEW, CEZ, RM and JR) agreed to extract data from the survey/questionnaire data from mixed method studies to avoid missing any qualitative data of importance, as well as interview quotations and author interpretations.

### Quality appraisal

Included studies were assessed on their methodological quality using the JBI Critical Appraisal Checklist for Qualitative Research.^23^ This checklist provided a framework for scoring the quality of studies by addressing particular aspects of research: for example, congruity between methodology, philosophical perspective, research objectives, means of data collection and analysis, researchers’ influence through interpretation and cultural orientation, and ethical considerations. A quality appraisal assessment of the included studies was not conducted to remove studies based on low quality, but to notify readers when interpreting findings that particular studies may show weakness in credibility and be less valid in accordance with the findings,.^24^ The first reviewer (AEW) independently assessed the methodological quality of all included studies, while the second reviewer (AK) assessed (15%) the studies independently. After discussion, assessment of the included studies was agreed.

### Data synthesis

This review used a thematic synthesis approach.^20^ This approach was to allow for the review to bring together findings beyond primary studies using an interpretive approach. Data analysis and synthesis consisted of three stages conducted by AEW: stage 1 involved line by line coding of the extracted data from the findings section of each study using the qualitative analysis software NVivo V.12 to create ‘free codes’; stage 2 involved the generation of descriptive themes, whereby similarities and differences within codes were assessed to form groupings of codes that created themes close to the original findings in the primary studies; the final stage, generation of analytical themes, was then conducted to go beyond the original findings and develop additional concepts relating specifically to the review question. Three online workshops with a group of four patient and public involvement (PPI) fathers (first time (n=2) and experienced (n=2), aged 27–39 years, with different backgrounds, including social economic status/ethnicity) were also conducted, whereby fathers contributed to stage 1, coding of included data, and stages 2 and 3, by aiding the development of descriptive and analytical themes. A data meeting with the research team (AEW, CEZ, RM and JR) was also carried out to help this development, discussing themes that were emerging and contributing to the overall analytical themes that specifically aligned with scoping review.

### Patient and public involvement

Contact with PPI fathers was made through a poster advertisement shared online within Newcastle University and the National Institute for Health and Care Research Applied Research Collaboration North East North Cumbria (NIHR ARC NENC) networks. Fathers provided feedback and added their personal interpretation as experienced fathers, while consulting within the thematic synthesis development (see table 2 for further details on PPI involvement). Feedback and insight from discussions with fathers provided a different perspective to researchers, placing more importance on specific concepts in the data that were not considered as important. This was shown when fathers coded differently or emphasised the importance of particular topics relating to the review aim, such as isolation within the perinatal journey, informing analytical theme development and shaping the reporting of the synthesis.

**Table 2:** Level of involvement at each patient and public involvement stage based on the ACTIVE continuum involvement table. Two levels of involvement were chosen over the selected stages.

Level of involvement	Stage
Influencing:stating, commenting, advising, prioritising or reaching consensus. Providing data or information that directly influenced the review process, but without direct control over decisions or aspects of the review process	Stage 1: collection of data (September 2022)Fathers were sent extracts of qualitative data from two selected papers within the review selection and directed on how to produce descriptive codes and notes. Fathers were asked to talk about what they interpreted from the data within a group, and a code spider diagram was created. These additional codes were inserted into the final codebook that informed analysis
Contributing:providing views, thoughts, feedback, opinions or experiences. Providing data or information that may indirectly influence the review process	Stages 2 and 3: analysis and Interpretation of findings (October 2022)Fathers were asked to relate data together, constructing linkages between codes from the codebook, and starting to develop categories that could further contribute to the development of descriptive themes. Discussions between fathers were further initiated by asking each of them to explain what categories they had created and the reasoning behind these categories. With fathers' contributions, a final thematic tree of descriptive themes, including researchers’ descriptive themes, was developed. Further broken down questions relating to the review question were asked to help guide fathers' thought processes when contributing to the development of analytical themes. Insight from fathers contributed to specific analytical themes, including theme 3, forgotten entity within the perinatal experience’, focusing on isolation

Research recommends incorporating PPI, within the later stages of a review (data collection and analysis and interpretation of findings), to add purpose, value and relevance to the review and public.^25 26^

## Results

**Figure 1:**
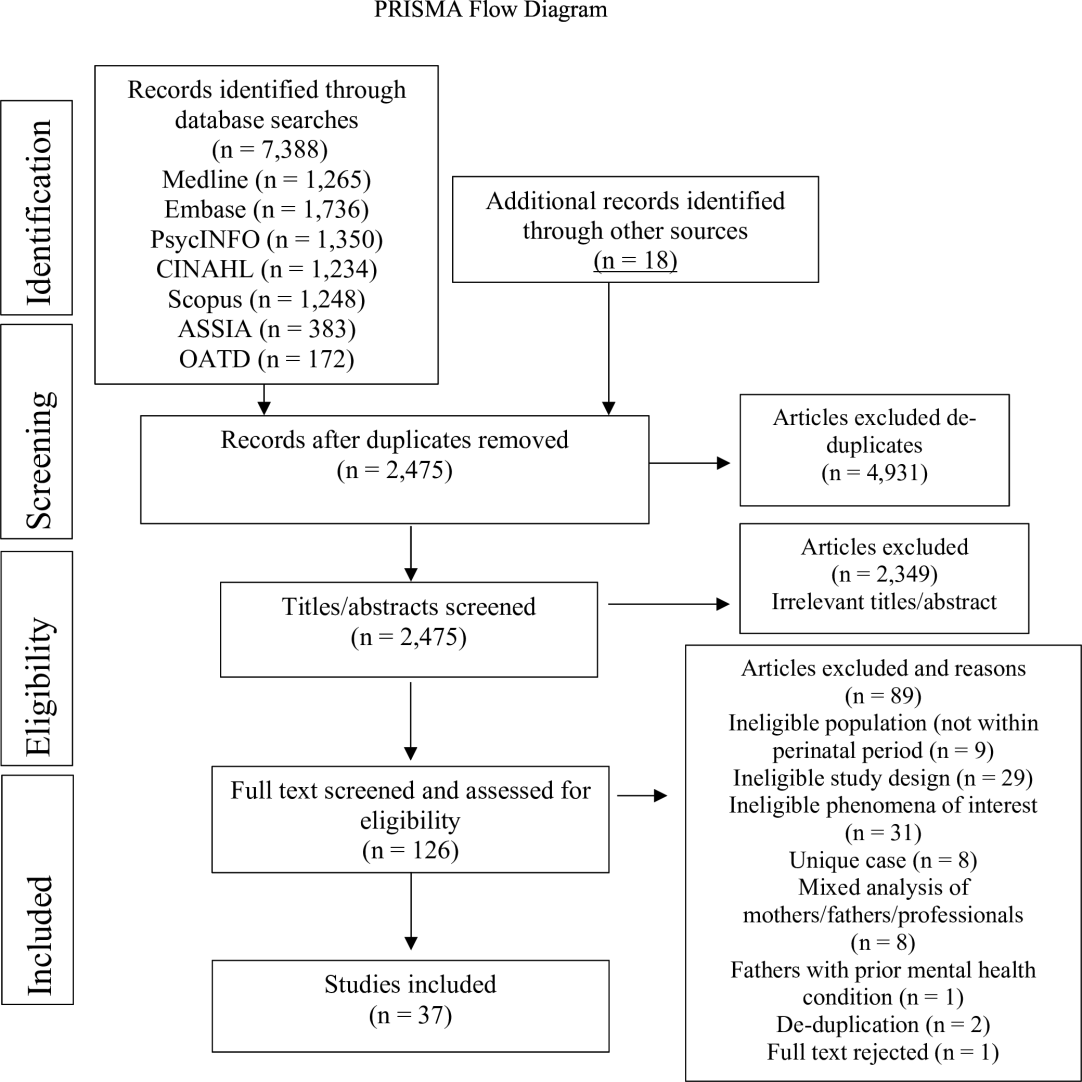
Preferred Reporting Items for Systematic Reviews (PRISMA) flow diagram showing the flow of studies through the scoping review.

Across the six databases and other sources, after de-duplication, 2475 studies were identified. Figure 1 shows the PRISMA flow diagram of the flow of studies.^27^ After full text screening, 37 studies were included.

### Characteristics of included studies

Most studies were conducted in the UK (n=11), Europe (n=9), Australia (n=7) and Asia (n=6) (four were from Singapore, one from Hong Kong and one from the Philippines), followed by the USA (n=1), Canada (n=1) and the Middle East (n=2). Included studies explored first time fathers’ experiences alone (n=21) and first time and experienced fathers’ experiences (n=7); the remaining studies were unclear or did not specify (n=9). Most studies included qualitative methods, such as in-depth interviews, focus groups and phone calls with fathers (n=33), with four studies using a qualitative online survey and questionnaire responses.

A total of 646 fathers participated in interviews/focus groups/phone calls and 1005 fathers provided a qualitative response within online surveys and questionnaires over the 37 included studies. Most studies used an inductive thematic analysis approach (n=14), with some studies employing interpretive phenomenological approaches (n=8), grounded theory, (n=5), content analysis (n=6), framework analysis (n=2) or narrative analysis (n=1), or others (n=1) did not specify their theoretical approach. Of the 37 included studies, studies focused on exploring fathers’ mental health and well-being experiences within the transition to fatherhood (n=30); the remaining studies focused on exploring fathers’ experiences of antenatal programmes/classes, interventional support and support helplines (psychological and social) within the perinatal period (n=7) (table 3 has the full data extraction of the included studies).

**Table 3:** Summary of included studies (n=37). Modified Joanna Briggs Institute data extraction tool was used

Author, year	Methodology	Method	Phenomena of interest (aim)	Sample size/participants	Setting/city/country	Data analysis
Baldwin *et al*, 2019^4^	Qualitative	Semi-structured interviews. Postnatal	To develop an understanding of men’s experiences of first time fatherhood, their mental health and well-being needs	21 first time fathers with children <12 months.Ages 20 to >60 years (10 Indian, seven white, 1 Spanish, 1 Black African, 1 Black Caribbean, 1 Pakistan, very highly educated)	National Health Service London boroughs UK	Framework analysis
Baldwin *et al*, 2021^28^	Mixed methods	Online pre/post questionnaires, and semi-structured telephone interviews. Antenatal and postnatal	To test the feasibility and acceptability of the Promotional Guide system with first time fathers and pilot potential outcome measures to assess their mental health and well-being	10 first time fathers were interviewed for the qualitative section (highly educated, mix of ethnicities 10 interviewed not specifically stated)	UK London NHS Organisations	Framework analysis
Baral and Guzman, 2021^29^	Mixed methods	Structured interview questions.Postnatal	To understand the anxieties and coping of new fathers with postnatal depression	7 first time fathers with postnatal depression. Few weeks after birth(all but one employed)	Selected from 10 barangays, Cabanatuan City, Philippines	Grounded theory analysis
Barclay *et al*, 1996^30^	Qualitative	Focus groups. Antenatal	This paper reports on research conducted with men who were attending antenatal classes with their partners who were pregnant for the first time	53 first time fathers (75% Australian, mainly higher education)	Australia 2 Sydney Hospitals1 community health centre	Grounded theory analysis
Dallos and Nokes, 2011^31^	Qualitative	Semi-structured interviews. Postnatal	This study attempts to map the experience of fathers following the birth of a child	5 First time fathers. (white British, mixture of professionals)	UK Parenting Groups	Interpretive phenomenological approach
Darwin *et al*, 2017^15^	Qualitative	Semi-structured interviews. Postnatal 5–10 months	Fathers’ views of their own mental health experiences within the perinatal period	19 first time and subsequent fathers. Aged 25–44 years. Baby 0–12 months. (18/19 white British, highly educated)	Yorkshire UK BaBY epidemiological cohort	Thematic analysis
Deave and Johnson, 2008^49^	Cross sectional study	Semi-structured interviews in pregnancy and 3–4 months postnatal	To explore the needs of first time fathers in relation to the care, support and education provided by healthcare professionals during the antenatal period	20 partners of women first time fathers (all white British except 1 Asian and 1 Brazilian)	UK South West EnglandHealthcare provider	Content analysis
Edhborg *et al*, 2016^19^	Qualitative	Semi-structured interviews3–4 months postnatal	To describe fathers’ experiences of the first year postpartum, when they showed depressive symptoms 3–6 months postpartum	19 first time fathers (17 Swedish, 1 non-Swedish, 15 higher education)	Sweden, Stockholm	Qualitative content analysis
Fagerskiold 2008^50^	Qualitative	Interviews. Postnatal	To explore first time fathers’ experiences during early infancy of their children	20 first time fathers aged 20–48 years (mainly higher education)	Southern SwedenRegional social insurance offices	Grounded theory
Ngai and Lam, 2020^51^	Qualitative	Semi-structured interviews. Postnatal	To explore men’s experience of first time fatherhood and coping in Hong Kong	44 first‐time fathers (all Chinese, 2 main caregivers)	Hong Kong, China	Thematic analysis
Fenwick and Bayes, 2012^55^	Qualitative	Two semi-structured interviews. Prenatal and postnatal	To explore and describe men’s experiences of pregnancy and childbirth expectations	12 expectant fathers (first time/experienced not reported).Interviews took place during pregnancy and after childbirth	Australia Australian Teaching Hospital	Thematic analysis
Finnbogadottir, *et al*, 2003^32^	Qualitative	Narrative interview form and qualitative content text. Antenatal	To describe first time expectant fathers ‘experiences of pregnancy	7 first time and expectant fathers (all Swedish, varied educational levels)	Sweden Antenatal Clinic	Content text analysis
Fletcher *et al*, 2020^54^	Mixed methods	Phone calls with counsellors (time period not specified)	The present study examines fathers’ contacts with the Perinatal Anxiety and Depression Australia (PANDA) National Helpline	129 fathers were included within the phone call service (first time/experienced not reported)	Australia (PANDA) National Helpline	Thematic analysis
Gottfredsdottir, 2005^56^	Qualitative	Focus group interviews. Weeks 27–37 gestation	The objective of the study was to explore prospective first time fathers' views concerning fatherhood in relation to this new emphasis on their right to parental leave	15 first time fathers. Average age 24 years (all mixed social class and education level)	Iceland Reykjavik Antenatal Clinic	Thematic analysis
Hall, 1994^33^	Qualitative	Semi-structured interviews. Postnatal	Explore fathers process of redefining their roles as spouses, workers and fathers	10 first time fathers in dual earner families (8 Caucasian and 2 Chinese Caucasian)	Canada Through occupational nurses	Grounded theory analysis
Hodgson *et al*, 2021^34^	Qualitative	Semi-structured interviews. Postnatal	To explore men’s transition to fatherhood	12 first time fathers with a child <2 years (11 white British and 1 white other)	UK Sheffield University Research	Grounded theory analysis
Johannsson *et al*, 2016^35^	Mixed methods	Self-report questionnaires. Antenatal and postnatal	The aim was to explore what concerns Swedish fathers had about parenting difficulties at 2 months after the birth of their baby	827 fathers (first time/experienced not reported). (Majority Swedish, mainly highly educated)	Sweden Prospective Longitudinal Cohort Study	Content analysis
Johansson*et al*, 2020^36^	Qualitative	Interviews. Postnatal	The study aims were to explore the lived experiences of mothers and fathers of postpartum depression and parental stress after childbirth	5 fathers (first time/experienced not reported). Aged 30–40 years (highly educated)	Sweden Child Health Centre	Interpretive phenomenological approach
Kowlessar*et al*, 2014^37^	Qualitative	Interviews. 7–12 months after birth	The objective of this study was to explore the experiences of fathers during their first year as parents to fully capture their experiences and transition to parenthood	10 first time fathers (all white British)	UK Manchester Antenatal Classes NHS	Interpretive phenomenological approach
Lagarto and Duaso, 2022^38^	Qualitative	Semi-structured interviews. Antenatal	This study explored fathers’ experiences of paternal–fetal attachment	10 fathers to be (first time/experienced not reported) (8 white European, 2 BAME background). Aged 29–40 years	UK London NHS	Interpretive phenomenological approach
Ling, Cheng, Shorey (2021)^39^	Qualitative	Semi-structured interviews. Postnatal	The aim of this study was to explore distressed fathers’ experiences and needs in the early postpartum period	12 fathers (not restricted to first time fathers), aged ≥21 years (Chinese, Malay and Indian)	Singapore Public Hospital	Thematic analysis
Machin, 2015^40^	Mixed methods	Semi-structured interviews 6 months postnatal	Study aims to explore the experiences of first time fathers	15 first time fathers (12 white British, 1 white other, 1 mixed white, 1 black Caribbean, 1 Indian). Higher education	UK OxfordshireNational Childbirth Trust	Type of analysis unknown
Nesporova, 2019^41^	Longitudinal qualitative	Interviews. 2 in pregnancy, 1 postnatal	The study focuses on the transition to fatherhood and the life changes which fatherhood effects in men’s everyday lives	16 fathers (first time/experienced not reported). (All European)	Czech Republic International TransPARENT Project	Comparative thematic analysis
Palsson *et al*, 2017^42^	Qualitative	Interviews 1 month after baby was born	To describe first time fathers’ experiences of their prenatal preparation in relation to challenges met in the early parenthood period	15 first time fathers,19–37 years (12 European,1 Macedonia, 1 Iran, 1 Sri Lanka). Mixed education levels	Southern Sweden postnatal units	Interpretive phenomenological approach
Pedersen *et al*, 2021^57^	Qualitative	Semi-structured interviews. Postnatal	This study aimed to explore the lived experiences of men or fathers with paternal perinatal depression and to understand the barriers and facilitators of help seeking among fathers with paternal perinatal depression	8 fathers with paternal perinatal depression (first time/experienced not reported).(All Danish citizens)	Denmark CopenhagenPregnancy and Paternity Groups online	Interpretive phenomenological approach
Rayburn *et al*, 2021^60^	Mixed methods	Open ended qualitative responses from satisfaction surveys. Antenatal and postnatal	The study examined the feasibility and acceptability of Becoming Fathers, a brief intervention for expectant and new fathers that combined education and self-care skills like mindfulness in a supportive group format	6 fathers first time and expectant completed whole study, started with 21	USA Obstetric Clinics and Childbirth classes	Thematic analysis
Rominov *et al*, 2018^53^	Qualitative	Semi-structured face to face. Postnatal	To explore men’s experiences of seeking support for their mental health and parenting in the perinatal period and identify their specific support needs during this time	20 fathers (first time/experienced not reported) who were expecting or parenting an infant <2 years. Mainly Australian	Australia University Research	Content analysis
Shorey *et al*, 2017^43^	Qualitative	Semi-structured interview. Postnatal	To explore first time fathers’ postnatal experiences and support needs in the early postpartum period	15 first time fathers (Chinese/Malay/Indian, highly educated)	Singapore Public Tertiary Hospital Postnatal Wards	Thematic analysis
Shorey *et al*, 2018^58^	Qualitative	Interviews. 6 months postnatal	This study aims to understand fathers’ expectations, needs, and experiences in infant care during the early postpartum period in Singapore	50 first time and experienced fathers (Chinese/Malay/Indian, highly educated)	Singapore Postnatal Wards Public Hospital	Thematic analysis
Shorey *et al*, 2019^44^	Qualitative	Semi-structured interviews. 6 months postnatal	This study aims to understand paternal involvement within the 6 month postpartum period to identify the challenges and needs of Singaporean fathers	50 first time and experienced fathers (varied ethnicities)	Singapore Postnatal Wards Public Hospital	Thematic analysis
John *et al*, 2005^52^	Qualitative	In-depth audiotaped interviews6–12 weeks after birth	To explore fathers’ perspectives on the experiences, processes and life changes in the early weeks of fatherhood	18 first time fathers (western/European, highly educated)	Australia QueenslandPublic Hospital postnatal wards	Thematic analysis
Tehrani *et al*, 2015^59^	Qualitative	Individual open ended interviews 32 −40 weeks of pregnancy	The purpose of this study was to explore how first time fathers describe their experiences of pregnancy	26 Iranian and Moslem first time fathers (highly educated)	Iran Tehran Public Health Prenatal Care Clinics	Qualitative content analysis
Wilkes *et al*, 2012^45^	Qualitative	In-depth interviews. Antenatal	To explore the experiences of prospective adolescent fathers regarding their impending fatherhood	7 adolescent expectant fathers (first time/experienced not reported) (white European, 3/7 unemployed, aged 16–22 years)	Australia Antenatal Clinic at Maternity Centre	Narrative analysis
Clifford-Motop *et al*, 2022^46^	Qualitative	Semi-structured interviews. Postnatal	This qualitative study explores the experiences and perceptions of new and expectant First Nations fathers in an urban setting in Australia	8 First Nations fathers first time and experienced, 18–33 years,5/8 unemployed	Australia Substudy Indigenous Birthing	Descriptive phenomenological analysis
Davenport and Swami, 2023^47^	Qualitative	Semi-structured interviews. Postnatal	To explore postnatal depression experiences among fathers in the UK	8 fathers UK, first time and experienced fathers, 27–41 years,7 white British, 1 Asian	UK University research	Interpretive phenomenological approach
Kaner, *et al*, 2023^61^	Cross sectional study	Session transcripts and 2 year post-intervention surveys. Postnatal	This study explores an innovative online group intervention for new fathers, focusing on participation experience of fathers in the online programme	122 first time Israeli fathers. Median age 33 years. Highly educated	Israel Online Discussion Group	Thematic analysis
Reay *et al*, 2023^48^	Cross sectional study	Free text response questions. Postnatal	This research explores the barriers fathers face to seeking help for paternal perinatal depression	50 fathers first time and experienced fathers, 18–65 years	UK Online Qualtrics survey	Thematic analysis

### Quality appraisal findings

Studies were reported in accordance with the JBI criteria. Six out of the 37 studies scored nine out of 10 on the JBI checklist, of the remaining studies 14 scored eight out of 10, 16 scored seven out of 10 and one study scored 5 out of 10 (see online additional file 2 for JBI Critical Appraisal Table). For most studies, there was congruity between the methodology, research question/objectives, and representation of data, as well as ethical approval and use of qualitative quotes. However, the majority of studies under-reported the researcher’s cultural and theoretical orientation or influence of researcher interpretation. Many of the studies reported the qualitative methodology, however 12 studies did not state the philosophical orientation on which the study was based, this can be considered less rigorous in design. These studies were still considered to show moderate to high levels of quality appraisal; according to the JBI criteria ranging between 5 and 10, no studies were removed.

### Synthesis findings

A thematic synthesis of the 37 included studies was conducted to explore fathers’ mental health and well-being concerns, as well as experiences of antenatal programmes and psychological and social support, in addressing these concerns.^21^ Generation of descriptive themes from codes resulted in six overarching themes and 15 subthemes incorporated within a thematic map. Development of analytical themes that went beyond the original findings and themes from individual included qualitative studies, presented additional concepts specifically relating to review question, concluding four themes and eight subthemes.

The following themes were identified: diminished partner relationship; provider or protector? multiplicity of fatherhood identification; forgotten entity within the perinatal experience; and "I try to battle it myself," masculine ideals within fatherhood. These synthesised themes and subthemes are presented below in table 4 (see online additional file 3 for table showing which individual studies relate to each theme/subtheme). Each theme, including subthemes, are described below accompanied by illustrative quotes to provide evidence (table 4).

**Table 4:** Synthesised themes

Synthesised theme	Synthesised subtheme	Quote
Theme 1: Diminished partner relationship(Objective a)(Objective a)	Invasion of infant Perinataladjustment demandsMy child is my partner’s child Inequality in parenthood from mother	“We don’t usually argue, we don’t snap at one another. And…knowing I was doing it…for no good reason was really upsetting.” (Father, UK)^49^ p.631“I enjoy getting involved with it, but … my partner … tends to take over … she seems to feel that she’s the mother … that I can’t do it properly.” (Father, UK)^31^ p.157
Theme 2: Provider or protector? Multiplicity of fatherhood identification(Objective a)(Objective a)	Balancing culturally specific fatherhood expectations Parental confrontation from generation gap	"I would say that’s the largest part I have to play, lah, to really ﬁnancially support the whole household… Ya, I guess that’s about it, lah.” (Father, Singapore)^44^ p.9 “My mother-in-law and I had a tension regarding breastfeeding. She didn’t breastfeed in the past and pushed the same for my baby. So, I mean, I was quite insistent, to the extent of like quarrelling with her.” (Father, Singapore)^58^ p.32
Theme 3: Forgotten entity within the perinatal experience(Objective a)(Objective b)	Exclusion and degrading humour from healthcare professionals Neglection of fatherhood education as a toolkit	“Everybody I’ve been in contact with has, … sort of, been in the mindset of treating you like you’re a bit of a tool… I was a dad putting the poppers on in a room full of mothers, they’re (health professional) like ha, ha, you know, look, dad’s struggling.” (Father, UK)^4^ p.8 “But it was none of the kind of, practical tips of what to do once things start going wrong, in the sense that your child may not know how to latch. So, as a dad, what can you do to, kind of, support that?” (Father, UK)^4^ p.7
Theme 4: "I try to battle it myself," masculine ideals within fatherhood(Objective b)(Objective a)	Isolation of emotional struggles "Is this normal?" Desperate for validation from peers	“But I suppose as a man I think … it’s always been a perception that we’re supposed to able to handle it.” (Father, UK)^31^ p.158 “We blokes are rubbish at talking.” (Father, UK)^4^ pg.7

#### Theme 1: diminished partner relationship

##### Invasion of infant, perinatal adjustment demands

Across 24 of the included studies, it was clear that fathers had many concerns within the transition to fatherhood due to the perinatal changes and demands of adjusting to an entirely different lifestyle.^415 19 28^,^48^ A particular concern highlighted throughout 18 studies was the initial loss and change to their romantic relationship with their partner, which impacted fathers’ overall mood and well-being.^415 29^,^33 36 39 42^ The overwhelming demands within the perinatal adjustment included exhaustion and tiredness from caring for an infant and new responsibilities, leaving little time for leisure and intimacy: “my wife and I hardly ever talked, we just looked after our son” [Father, UK]^46^ p.1195. Certain fathers within six of the included studies were seen to pinpoint exhaustion on the infant specifically portraying them as a catalyst for confrontation and lack of interest with their partner.^4 19 31 33 43 44^ Fathers were unaware of possible changes to their relationship dynamic after and expressed the need for support in mentally adjusting and dealing with this change, as stated by one father,

“Would have been good to know that support that would be there for a father that has had his wife/girlfriend to himself for so long and then all of sudden they've got a baby that shifts the attention … I had a bit of an issue with it.” [Father, UK]^53^ p.462

Fathers in 15 studies also expressed that they would argue about insignificant dilemmas and often found that their partners unappreciated their involvement during the perinatal period.^415 19 29^,^31 39 43 45^ One father specifically stated, “I ask her every day, do you want me to be around and shit…well stop putting me down” questioning their fatherhood value [Father, UK]^46^ p. 1940. These fathers struggled to understand and comprehend this new confrontation, igniting alterations in mood and increased frustration with each other, but mainly with themselves. The impairment of relationship was an explanation for depressed mood and poor emotional well-being as shown below,

“We don’t usually argue, we don’t snap at one another. And…knowing I was doing it…for no good reason was really upsetting.” [Father, UK]^49^ p.631

Sometimes fathers’ anger was directed towards their partner “a lot of the time I was angry at people like my wife”, as well as expressing concerns that their feelings towards their partner were out of control and talked of *“*punching the wall*”* when describing aggressive exchanges [Father, UK]^47^ p.1193 and [Father, Australia]^51^ p.7. For most fathers captured in 10 studies, this lack of quality time together, over-exhaustion and heightened negative emotions, due to transitional parenting demands, created a division and crack between them, developing concerns of a disconnected relationship.^419 29^,^32 43 46 47 54^ This contributed to impacting not only the fatherhood transition negatively, but also the mothers and possible future childhood psychological development.

##### “My child is my partners child,” inequality in parenthood from mothers

Fathers also expressed concerns relating to not feeling part of the relationship with the mother and infant, primarily due to isolation as a result of the mother disallowing their involvement, often referred to as maternal gatekeeping within the literature.^1519 31^,^35 37^ Across five studies, fathers initially felt left out of the pregnancy as it was viewed as a “physical experience unique to their partners”, making them feel as though they were “standing beside the pregnancy” or “secondary” [Father, Australia]^55^ p.6, [Father, Sweden]^32^ p.100 and [Father, Australia]^46^ p.1940. Mothers were then seen to present heightened protectiveness of the child and had differing expectations to fathers within the postnatal period. Fathers viewed mothers as taking on more stereotypical roles, influencing inequality within parenthood, that recent fathers desired to distance from, as stated,

“I enjoy getting involved with it, but … my partner … tends to take over … she seems to feel that she’s the mother … that I can’t do it properly.” [Father, UK]^31^ p.157

Participants in 11 studies reported how these experiences had the effect of causing them to feel frustrated towards their partner, as well as develop self-doubt and concerns about being a good father.^1519 31^,^33 37 38 46 50 52 54 56^ One father stated feelings of “I am less worth, and I am a bad parent”, [Father, Sweden]^19^ p.434. Support and admiration were sometimes sought from partner in their role as father, however, instead they felt neglected and unsupported,

“She’s never, never given me praise as a dad (crying), which is upsetting as you can see, but, cos, you want to be good enough …” [Father, UK]^31^ p.156

Certain fathers across six studies often feared that due to their belief/view of an innate mother–infant/biological bond that they lacked within the antenatal and postnatal period, they would find difficulty in forming a bond with their child.^15 37 38 44 52 54^ Fathers experienced much anxiety when they did not fall in love immediately, placing emphasis on their partner hindering their involvement and worsening their fears of rejection: “I haven’t experienced feeding until recently, I wouldn’t say I was jealous of my wife, but resentful…she was able to calm him and I couldn’t” [Father, UK]^40^ p.45. These non-equal parenthood attitudes and behaviours directed from partners associated with not building a healthy bond with the child only contributed towards self-doubt and distress as well as resentment towards their partner, influencing transition process negatively.

### Theme 2: provider or protector? Multiplicity of fatherhood identification

#### Balancing culturally specific fatherhood expectations

Fathers reported in 17 of the included studies experiencing confusion about what was expected from them as a father within society.^415 19 29^,^35 37 46 47 49 50 55^ Many of these fathers struggled to adopt a role when becoming a father due to the multiplicity of whether they should be adopting a more traditional providing parenting approach, or a more modern care giving protecting parenting approach, challenging prevailing stereotypical roles [Father, UK]^15^ p.8. Some fathers in eight included studies portrayed a strict mindset of providing financial support to their family as their fatherhood role, referring to themselves as the "breadwinner…to earn a living" [Father, UK]^47^ p.1195.^39 42 43 46 47 51 58 59^ Others placed more importance on providing financially, as well as supporting and spending more time with their child and partner, adopting equal parenthood roles, as shown by one father,

“In parenthood everything is joint, I don’t believe I should be the sole money earner and my partner should be the sole parent … (our job) is to provide with money, emotional support, protection, love, everything.” [Father, UK]^40^ p.42

An attempt to balance the multiple roles of father, employee and husband led most of the fathers in 21 studies within the review to develop unrealistic expectations for themselves, referring to the transition as “non-stop-ness of stress,” in time draining themselves physically and mentally [Father, UK]^15^ p.5.^415 19 30^,^40 42 46 47 49 54 56 57^ In almost all of the included studies, fathers experienced concerns over financial threats, which appeared strongly related to bouts of anxiety.^415 19 28^,^41 45 47 49^ Although many of these fathers, in six of these studies, feared that they may be ‘missing out’ when at work, when required to work even longer hours, this led to further distress and worry of fear of rejection from the infant.^15 31 38 52 54 57^ For example, “it was an hour yesterday, which I felt wasn’t sufficient connection” [Father, UK]^31^ p.158.

The perceived multiple demanding societal expectations of a father were however culturally specific, mainly portrayed within westernised cultures within this review. Westernised fathers included in this review appeared to be adopting a much more modernised parenting approach, expressed across 13 studies.^4 15 19 31 33 35 36 40 42 46 50 56 57^ On the other hand, many non-westernised or eastern fathers in four of the included studies still heavily adopted a more traditional parenting breadwinner approach.^43 44 58 59^ These fathers felt that they did not need to adhere to multiple roles, possibly due to different cultural expectations and religious beliefs. They stated that “children rising represented the mothers’ duty” and that the “paradise is under the mothers’ feet,” highlighting the importance of the mother’s role in this period [Father, Iran]^59^ p.3. This contrasted with fathers’ duty of obtaining higher income to lead to “peace in the family” and “development of love” [Father, Iran]^59^ p.4. This contributes towards more realistic expectations within fatherhood and lessened negative mental health experiences.^43 44 58 59^

“I would say that’s the largest part I have to play, lah, to really ﬁnancially support the whole household… Ya, I guess that’s about it, lah.” [Father, Singapore]^44^ p.9

A few fathers from two Singapore studies did however express beliefs to start moving away from culturally traditional parenting styles: “traditional Chinese confinements…it’s not good for my wife and child, different generations will have different views” [Father, Singapore]^43^ p.6.^43 44^ More non-westernised or eastern fathers may be seen to alter their approaches in time, attempting to balance these multiple roles of provider and protector and potential stresses.^43 44 58 59^ When exploring fathers’ concerns from differing cultures, across these included studies, it is considered that fathers do not have similar concerns regarding balancing multiple roles and unrealistic expectations impacting mental health and well-being negatively within the transition to fatherhood.

#### Parental confrontation from generation gap

Fathers’ confusion over what the fatherhood role and identity involved was heavily influenced by parents' and in-laws' expectations and experiences. Advice from this generation tended to point fathers towards displaying a more traditional fatherhood parenting approach. Westernised and non-westernised fathers in 12 of the included studies expressed concerns regarding confrontation with parents and in-laws due to this so-called generation gap, disagreeing on how to raise their child.^4 15 19 29 31 38 39 43 45 49 52 53 58^ This conflict and criticism contributed towards disputes, disagreements and additional stress for fathers, impacting their mood negatively as well as their transition process.

“My mother-in-law and I had a tension regarding breastfeeding. She didn’t breastfeed in the past and pushed the same for my baby. So, I mean, I was quite insistent, to the extent of like quarrelling with her.” [Father, Singapore]^58^ p.32

As shown above, quite often advice from parents and in-laws mirrored their own childhood experiences, but some (particularly westernised) fathers across six studies actively voiced wanting to avoid a repeat of their own upbringing: “I just want to be a father that I never had” [Father, UK]^46^ p.1942.^33 38 45 46 49 56 57^ They showed an interest in being more present with their child, adopting a more modern parenting approach, but also wished to show more emotional tendencies and support, distancing from masculine ideals and lack of support related to their own childhood,

“I always want my children to be … able to laugh with me and always be able to come to me if they are feeling sad … (my father) wasn’t very forthcoming about his feelings.” [Father, UK]^38^ p.334

On the other hand, non-westernised/eastern fathers across five included studies were still seen to actively seek advice and guidance from their parents and in-laws.^43 44 51 58 59^ They viewed the importance of support and “co-ordination with the family” positively and mainly reassuring in supporting their mental health [Father, China]^51^ p.727. These fathers suggested little if any parental confrontation or generation gap, and therefore, not expressing similar concerns regarding parental confrontation.

### Theme 3: forgotten entity within the perinatal experience

#### Exclusion and degrading humour from healthcare professionals

In 19 included studies, a key concept acknowledged within the data was the vast concern from fathers regarding isolation and exclusion during the perinatal experience as a whole, specifically the concern of physical and verbal exclusion from healthcare professionals.^415 28 30 32 34 36 38^,^40 43 46 48 49 51 53 55^ Fathers stated that professionals isolated them from perinatal activities in the antenatal and postnatal periods in consultation rooms (“the midwife drew the curtain around me”) and within the birthing experience: “was pretty much like I didn’t exist” [Father, UK]^40^ p.52 and [Father, Australia]^55^ p.6.

Across seven studies there was heightened importance from fathers in regard to witnessing antenatal appointments, including ultrasound scans and being involved in the early stages of pregnancy.^32 34 38 43 46 55 56^ Fathers felt that attending these appointments helped remove feelings of being in denial and no longer viewing the pregnancy as an “abstract entity” but rather proof of it being real [Father, UK]^34^ p.4. This helped fathers within the transition mentally, relieving anxiety and apprehension.

“Seeing the baby not only helped him ‘connect’ with his child but also allayed some of his anxiety.” [Author]^55^ p.6

Fathers experienced professionals express negative, degrading attitudes and humour towards themselves, while not acknowledging them.^4 15 28 30 32 34 38 40 46 48 53 57^ Fathers felt frustrated and angered due to this non-acknowledgement but specifically in regard to their general impoliteness as if they were invisible, as stated by one father,

“Everybody I’ve been in contact with has, … sort of, been in the mindset of treating you like you’re a bit of a tool… I was a dad putting the poppers on in a room full of mothers, they’re (health professional) like ha, ha, you know, look, dad’s struggling.” [Father, UK]^4^ p.8

This caused fathers to feel “unwanted and alienated”, being considered a “passenger” throughout the perinatal journey rather than actively participating: “I’m just down on the side listening” [Father, UK^28^ p.13 and [Father, UK]^46^ p.1939. This behaviour only added more insecurities as well as influencing their transition process negatively. On the other hand, certain fathers were not excluded by healthcare professionals, shown in one study whereby professionals justified the importance of feeling valued within their separate role as father; they were considered an “important piece of the jigsaw” [Father, UK]^4^ p.8.

#### Neglection of fatherhood education as a toolkit

A further concern acknowledged was the lack of importance in involving and educating fathers in parenting information and educational classes/programmes during the antenatal period. In 20 included studies, fathers particularly felt that they needed to be better informed on parenting in general, but especially breastfeeding issues and problem solving information in the antenatal period, that they could use as a toolkit for future preparation.^415 19 28 30^,^32 34 37 42^ A key concern from fathers was the unpreparedness of fatherhood and what to do when matters do not go to plan,

“But it was none of the, kind of, practical tips of what to do once things start going wrong, in the sense that your child may not know how to latch. So, as a dad, what can you do to, kind of, support that?” [Father, UK]^4^ p.7

Fathers had worries regarding their child’s health and employing “strict breastfeeding routines”, but when these routines did not go accordingly, they felt underprepared and clueless as to what to do [Father, Denmark]^49^ p.5. Quite often when these issues did arise, it was them as fathers who had to practically help in fixing the situation, but fathers did not feel well equipped or supported on how to amend these issues.^4 34 37 42 49 53 56 60^ This led to further decline in fathers’ mental health within the postnatal period.

“When our son was born, the breastfeeding didn't quite kick off. Hospital staff-couldn't you have just had a brochure or pamphlet, or as part of the classes to tell us that we should go and get all of that stuff beforehand just in case? That really would've taken a lot of the stress out of it. My wife was in no state to go to the chemist herself.” [Father, Australia]^53^ p.460

Fathers in nine included studies were also not satisfied with the extent of generic parenting information, feeling forgotten and isolated in informing them on correct vital information that was restricted to the mother alone.^4 34 37 42 49 53 56 58 60^ This was portrayed not only in classes/programmes, but also within the differing modes of presenting parental information, such as “Little grey boxes for the fathers, as if the man is only going to read the summary” [Father, Australia]^53^ p.457. Antenatal education/programmes are not being seen as a help to guide the transition to fatherhood, contributing to stress and impacting fathers within the postnatal period negatively.

### Theme 4: “I battle it myself,” masculine ideals within fatherhood

#### Isolation of emotional struggles

Crucially highlighted in over half of the included study findings, by fathers, was the need to deal with mental health concerns alone.^415 28^,^31 34 36 38 40 42 45^ Fathers across 26 included studies experienced heightened physical and emotional breaking points with bouts of exhaustion and postnatal depression symptoms within the perinatal period: “I could feel myself sort of slipping” [Father, UK]^47^ p.1192.^415 19 28^,^32 34 36 38^ However, fathers did not want to admit their emotional struggles regarding “domestic issues” [Father, UK]^49^ p.158. Paternal perinatal mental health overall was considered a taboo, to admit being distressed, with fathers stating that “as a dad you should not show weakness”, or that “it’s always been a perception that we’re supposed to be able to handle it.” [Father, UK]^48^ p.11 and [Father, UK]^31^ p.158. Theses masculine ideals and stereotypes had a major role in influencing fathers’ actions to not reach out, indirectly isolating fathers from emotional and mental health support.

Additionally, findings from over nine studies also provided insights into an apparent lack of fatherhood support, particularly relating to emotional/psychological formal support.^415 28 34 38 46^,^48 57^ General practitioners were considered “too stretched” in reviewing these concerns, often showing “lack of awareness of paternal perinatal depression,” and “specific paternal perinatal mental health therapists” were limited. [Father, UK]^4^ pg.8, [Father, UK]^48^ p.11 and [Father, Denmark]^57^ p.7. Fathers acknowledged that their importance of psychological/mental health hurt, and support was not on par with mothers, worrying about whether they “would be taken seriously” by professionals [Father, UK]^48^ p.11.

“I think it was about 6 weeks … and then (starts to break down) sorry I am very emotional … so yeah, surprisingly, yeah it was really good … sorry, I have not really sort of spoken about it, so, you will have to excuse me (voice breaking down).” [Father, UK]^38^ p.335

Paternal postnatal depression was acknowledged as a gender specific condition; this was seen to be due to information within the healthcare system restricted to mother inclusive terminology alone or a “lack of available advertised services for fathers” [Father, UK].^48 53 57^ Antenatal support and programmes were also seen to lack emotional insight towards fathers, being considered “not deep and meaningful” obverting addressing fathers’ mental health and emotional concerns relating to parenting [Father, UK]^4^ p.7. Evidently, commonly portrayed within this theme is the combination of the issue of masculine pressures and non-apparent fatherhood inclusive emotional support, causing fathers to continue to question their legitimacy in being entitled to mental health support.

#### “Is this normal?” Desperate for validation from peers

A key concern from fathers was the limited current support addressing emotions and mental/health concerns in the transition to fatherhood. In 12 studies, fathers reported the need for more opportunities to speak about these concerns, whether it was in classes or healthcare support, including “group therapy or counselling” [Father, UK]^48^ p.13.^4 15 28 32 34 38 46 48 53 57 60 61^ However, fathers in 10 studies expressed that peer support, including discussing and disclosing their experiences with friends and family in work and other social settings, were just as highly valued, due to “feeling more comfortable and relating” [Father, UK]^46^ p.1939.^1534 46 48^,^51 53 56 60^ They had concerns regarding what they were experiencing mentally during this period (altered behaviours and fluctuated mood), specifically whether they were regarded as "normal" responses.^3438 42 46 48^,^50 53 60 61^ They highlighted that talking to others about their experiences and making comparisons with work colleagues or in peer support group meetings was important in “finding comfort” and “validating” these experiences [Father, UK]^38^ p.335.^30^ While influencing their whole perception of fatherhood, this in time helped fathers to adjust their impractical and unrealistic expectations to more realistic fatherhood expectations: “You see that others go through the same thing, then you say, yes I’m normal, I’m average, I’m okay” [Father, Israel]^61^ p.5.

However, fathers emphasised that most of the time other fathers were not always willing to confide in each other regarding mental health and emotional concerns, shown in 10 included studies.^415 31^,^33 46 48 50 53 54 60^ Fathers primarily felt pressurised, again due to masculine ideals and societal stereotypes to avoid and overt these types of deep conversations and turn away from supporting each other, presuming it would not be helpful: “We blokes are rubbish at talking.” [Father, UK]^4^ pg.7. Acknowledgement that it is acceptable for men to talk about their parenting mental health concerns and positive peer support is fundamental in contributing to a more positive fatherhood transition.

## Discussion

This evidence synthesis of qualitative literature focused on fathers’ mental health and well-being concerns and challenges during the transition to fatherhood, as well as experiences of antenatal programmes and psychological and social support, to understand how we can better support fathers. Some fathers encountered positive experiences with no mental health repercussions, but most fathers in the review experienced many concerns throughout the perinatal period, impacting their overall mental health and well-being negatively. Fathers identified a paucity of paternal perinatal mental health support and educational programmes, that are not addressing current support needs. From a thematic synthesis of 37 included studies, across 12 countries, four themes were identified within the literature (table 4).

Consistently presented throughout these findings and in parallel with past studies was the alteration of fathers’ relationships with their partners as a main negative fatherhood adjustment, reported to impact fathers’ overall mood, well-being and personality.^62^ In this review, fathers expressed heightened emotional responses, including frustration and anger directed at partners during this period, as well as concerns regarding isolation of maternal responsibility from the mother. Previous research has shown that fathers can develop depression and negative emotions, such as anger and irritability around the birth of their child.^63^ The literature consistently also highlights how men who feel that there are problems of dyadic adjustment (changes in couple relationship functioning) are more likely to experience higher levels of paternal depressive symptoms.^64^ Mothers who are reported to show maternal gatekeeping behaviours are influencing inequality in parenthood and limiting fathers’ opportunities to participate.^65 66^ Research has established a link between fathers who have reported higher levels of depressive symptoms and higher levels of maternal gatekeeping, as well as having negative effects on parent–child relationships.^67^ Acknowledgement from mothers recognising their own gatekeeping behaviour and how this can impact fathers’ interactions and development with the child, as well as negatively affect mental health and well-being outcomes, may help contribute to more positive co-parenting experiences and restrict possible risks associated with negative mental health for fathers and mothers.^67^

Crucially highlighted within this review was confusion regarding what was identified as the fatherhood role; this undoubtedly led to pressures and stresses of balancing multiple roles, impacting their mental health negatively. Similarly, past research has reported associations between fathers’ negative mental health experiences and ‘role strain’, stress experiences due to expectations, and ‘role conflict’, emotional struggles experienced in balancing the demands of multiple societal roles.^9^ Further research has stated that both parents are more satisfied when there is a division of roles.^68^

Our understanding of operationalisation of fatherhood and father involvement has also changed over time.^69^ Therefore, different generations may have different advice and opinions relating to their societal experiences, leading to possible parental confrontation between family members, highlighted in this review, particularly with westernised fathers. This was shown whereby grandparents became a source of stress, with expectant couples experiencing conflict between the advice and information provided, contributing negatively to the fatherhood transition process.^70^ European and North American literature reports a tendency for fathers to be more involved in infant care than has been observed in past generations.^8^ It is considered that the ‘second demographic transition’ is possibly not as prominent in other cultures at present, compared with westernised cultures, with the perception of the meaning of fatherhood being different, leading fathers to adopt different paternity responsibilities.^59 71^ Further acknowledgement of confusion over societies' expectations of fathers and mental health implications, as well as associated emotional and practical antenatal preparation, may reduce fathers’ unrealistic expectations and reduce distress in the transition to fatherhood.^72^

It was also highlighted by fathers in the review that experiences of healthcare professionals’ intentional exclusion had caused them to develop the assumption that they are not entitled to be a part of the perinatal experience compared with the mother. This suggests the need for fathers to be equally respected by professionals during this period so as to influence their transition process more positively. In parallel, previous findings have shown fathers reporting eagerness and an intrinsic motivation to join their partner throughout the perinatal period, attending appointments, but often fathers received an unwelcoming environment from professionals.^73^

It was also apparent from the findings of this review that fathers felt excluded with regard to parenting information and education; this was especially relating to breastfeeding information and the concerns associated with not feeling prepared or equipped when breastfeeding did not go to plan. These unsupported breastfeeding experiences contributed towards a decline in their mental health. Fathers want structured parenting information, but find few interventions available with mainly poor insight.^12 73 74^ It can be considered that healthcare professionals have a key role in engaging fathers in education programmes and healthcare settings.^74^ Past research stated that fathers were more likely to attend antenatal appointments in the perinatal period, but these were often the first appointments to be cut due to low staffing.^75^ Further research conducted with health professionals exploring their perspectives on inclusion of fathers and acknowledgement of the importance of education and interventional fatherhood support may contribute to our understanding of whether possible inclusive training is needed for professionals or the provision of co-designed health information. Structured antenatal programmes focusing more on the emotional toll of fatherhood could help prevent poor mental health outcomes in the postnatal period, addressing specific possible future concerns.

Throughout the review, fathers expressed that they did not want to admit to their emotional issues but rather deal with them independently, conforming to masculine ideals. This is commonly shown throughout the literature worldwide, paternal perinatal mental health being considered a taboo.^31 76^ In the literature, and in accordance with this review, fathers have also stated a substantial lack of antenatal support, whereby they feel they can invest emotionally, as well as psychological/mental health postnatal support.^16 77^ This was highlighted throughout this review whereby a limited number of studies included initial subjective experiences of fathers attending specific antenatal or postnatal support/interventions.

It can also be considered that fathers are precluded from discussing the transition to fatherhood with their peers in support networks, feeling that they are not able to talk about their experiences freely and openly. This is due to gendered stereotypes and generational assumptions about pregnancy and fatherhood.^77^ Acknowledgement and building awareness that it is acceptable for men to talk about their parenting mental health concerns, by building on peer support groups within organisations, as well as further research on fathers’ experiences of current programmes/support and understanding fathers’ support needs, may contribute to the development of fatherhood tailored emotional and psychological/social support.

### Strengths and limitations

This work drew on multiple qualitative studies on a rising topic within research. This review included qualitative studies with varied qualitative methodologies, including methods of data collection, publication date, population or language. Therefore, a comprehensive search of all fathers from different backgrounds has allowed for a broader perspective. However, it is important to consider that although a total of 1651 fathers participated across the included studies, 1005 of these fathers took part in data methods, including questionnaires/surveys with qualitative comments. Therefore, it is likely that not all participant viewpoints were included in the findings of this review because they were previously summarised in primary studies.

To address the lack of interpretation specific to fathers’ experiences, being a female researcher, active involvement of a PPI group of four fathers over three workshops was incorporated within the analytical stages of the review. This helped with the overall validity of the conclusions and interpretations of the findings from a public perspective. However, when conducting critical appraisal of the included studies, it was agreed that studies would not be removed due to quality but reported in accordance with the JBI criteria. By using this method, studies were still included that may not have presented as valid findings due to the exclusion of qualitative requirements, such as lacking philosophical orientation in the methods. Half of the included studies in this review were published after previous systematic reviews, highlighting the importance of recent findings relating to fathers’ mental health concerns and experiences of up-to-date antenatal programmes, and perinatal psychological and social support within this review.

Most of the included studies were from westernised cultures (n=29), limiting the review findings to this population, as well as restricting global perspectives and cultural comparisons across (n=8) studies. Very few of the participants in these studies were from minority ethnic groups, with participants mainly being white British, highly educated (degree level and above) and aged >25 years. Further research is needed to explore the views and experiences of fathers under-represented within this review to assess for similarities or differences in findings for this target population. This review explored two objectives, the first objective exploring fathers’ mental health and well-being concerns/challenges within the transition to fatherhood, that was captured across six subthemes within the thematic synthesis. However, the second objective, exploring fathers’ experiences of antenatal programmes and psychological/social support, was only captured within two subthemes. This objective was only partially met due to insufficient studies available that captured this focus, limiting extracted relevant data and conclusions within the synthesis. There is a need for greater attention to fathers’ experiences of paternal perinatal support/programmes through research and practise to inform future interventional development. It is recommended review that further policy, practice and research implications are addressed. Recommendations are presented in box 1.

Box 1 **Policy, practice and research implications and recommendations**

**Practise:**

It is recommended that sufficient clinical guidelines are developed for the provision of paternal perinatal mental health support and management, drawing on the best available evidence. Health professionals should be sufficiently resourced to meet the needs of fathers, including provision of appropriate training, resources and time to provide inclusive emotional support for both parents, considering fathers as clients. Health providers should ensure adequate time is allocated to acknowledge fathers’ well-being during perinatal appointments, helping reduce stigma, normalise fathers’ responses, while providing an opportunity to sign post additional mental health support (including available resources and/or charitable organisations and services for fathers). Future recommendations include co-production, whereby fathers (who have experienced paternal perinatal mental health) are given the opportunity to contribute to healthcare/service decision making and co-designed health information.

Central and local government funding is recommended for charitable organisations and programmes who rely primarily on voluntary income to help set up support/peer groups for fathers reducing stigma.

**Policy:**

Healthcare policy makers and commissioners should recognise and seek to address fathers’ mental health and well-being within the perinatal period to support future service provision and father focused interventions.

**Research:**

Researchers are recommended to conduct research on fathers’ mental health and well-being experiences during the perinatal period from all backgrounds that are under-represented (socially and economically diverse, minority ethnicities, low income countries, non-English language). Further studies should report participant characteristics specifically so as to understand the different needs, so support can be optimised. Future research should focus on the development of effective and feasible, coproduced and inclusive interventions from evidence base that help support fathers’ mental health and well-being during the transition to fatherhood.

## Conclusions

This synthesis indicated that fathers found the transition to fatherhood difficult, compounded by insecurity within their role as both partner and father. Fathers found that there was a paucity of paternally focused support, not addressing fathers’ needs during the transition. By addressing fathers’ well-being concerns and challenges during the transition to fatherhood through the development of clinical guidelines on the management of paternal perinatal mental health, as well as effective practitioner/organisational engagement and inclusion of fathers, may assist in removing stigma and gender expectations that society still follows. It is clear that fathers require equal emotional and practical support to mothers during this period, and therefore it is important that future tailored support is provided and that fathers are not considered a ‘forgotten entity’, creating a more positive transition and parenting experiences.

## supplementary material

10.1136/bmjopen-2023-078386 online supplemental file 1

10.1136/bmjopen-2023-078386 online supplemental file 2

10.1136/bmjopen-2023-078386 online supplemental file 3

## Data Availability

All data relevant to the study are included in the article or uploaded as supplementary information.

## References

[1] Condon JT, Boyce P, Corkindale CJ (2004). The First-Time Fathers Study: a prospective study of the mental health and wellbeing of men during the transition to parenthood. Aust N Z J Psychiatry.

[2] Williams M (2013). Fathers reaching out. Midwives (1995).

[3] Chhabra J, McDermott B, Li W (2020). Risk factors for paternal perinatal depression and anxiety: a systematic review and meta-analysis. Psychology of Men and Masculinities.

[4] Baldwin S, Malone M, Sandall J (2019). A qualitative exploratory study of UK first-time fathers’ experiences, mental health and wellbeing needs during their transition to fatherhood. BMJ Open.

[5] NHS England, NHS Improvement, NHS Collaborating Centre for Medical Health (2018). The perinatal mental health care pathways. https://www.england.nhs.uk/wp-content/uploads/2018/05/perinatal-mental-health-care-pathway.pdf.

[6] Royal College of Psychiatrists (2011). Help is at hand – postnatal depression. https://www.sth.nhs.uk/clientfiles/File/PostNatalDepression%5B1%5D.pdf.

[7] Rowe HJ, Holton S, Fisher JRW (2013). Postpartum emotional support: a qualitative study of women’s and men’s anticipated needs and preferred sources. Aust J Prim Health.

[8] Genesoni L, Tallandini MA (2009). Men’s Psychological Transition to Fatherhood: An Analysis of the Literature, 1989–2008. Birth.

[9] Baldwin S, Malone M, Sandall J (2018). Mental health and wellbeing during the transition to fatherhood: a systematic review of first time fathers’ experiences. JBI Database System Rev Implement Rep.

[10] Dennis CL, Creedy D (2004). Psychosocial and psychological interventions for preventing postpartum depression. Cochrane Database Syst Rev.

[11] Mayers A, Hambidge S, Bryant O (2020). Supporting women who develop poor postnatal mental health: what support do fathers receive to support their partner and their own mental health?. BMC Pregnancy Childbirth.

[12] Charandabi SM-A, Mirghafourvand M, Sanaati F (2017). The Effect of Life Style Based Education on the Fathers’ Anxiety and Depression During Pregnancy and Postpartum Periods: A Randomized Controlled Trial. Community Ment Health J.

[13] Daley-McCoy C, Rogers M, Slade P (2015). Enhancing relationship functioning during the transition to parenthood: a cluster-randomised controlled trial. Arch Womens Ment Health.

[14] Carlson J, Edleson JL, Kimball E (2014). First-time fathers’ experiences of and desires for formal support: A multiple lens perspective. Fathering.

[15] Darwin Z, Galdas P, Hinchliff S (2017). Fathers’ views and experiences of their own mental health during pregnancy and the first postnatal year: a qualitative interview study of men participating in the UK Born and Bred in Yorkshire (BaBY) cohort. BMC Pregnancy Childbirth.

[16] Collins English Dictionary (2023). Westernized definition and meaning. collinsdictionary.com.

[17] Philpott LF, Leahy-Warren P, FitzGerald S (2017). Stress in fathers in the perinatal period: a systematic review. Midwifery.

[18] Philpott LF, Savage E, FitzGerald S (2019). Anxiety in fathers in the perinatal period: a systematic review. Midwifery.

[19] Edhborg M, Carlberg M, Simon F (2016). “Waiting for Better Times”: Experiences in the First Postpartum Year by Swedish Fathers With Depressive Symptoms. *Am J Mens Health*.

[20] Thomas J, Harden A (2008). Methods for the thematic synthesis of qualitative research in systematic reviews. BMC Med Res Methodol.

[21] Munn Z, Peters MDJ, Stern C (2018). Systematic review or scoping review? Guidance for authors when choosing between a systematic or scoping review approach. BMC Med Res Methodol.

[22] Joanna Briggs Institute (JBI) (2016). Data extraction tools. https://onlinelibrary.wiley.com/doi/pdf/10.1002/9781444316544.app3.

[23] Joanna Briggs Institute (JBI) (2016). Critical appraisal tools. http://joannabriggs.org/research/critical-appraisal-tools.html.

[24] Porritt K, Gomersall J, Lockwood C (2014). JBI’s systematic reviews: study selection and critical appraisal. AJN Am J Nurs.

[25] Pollock A, Campbell P, Baer G (2015). User involvement in a Cochrane systematic review: using structured methods to enhance the clinical relevance, usefulness and usability of a systematic review update. Syst Rev.

[26] National Institute for Health Research (2012). INVOLVE: public involvement in systematic reviews. https://training.cochrane.org/sites/training.cochrane.org/files/public/uploads/resources/downloadable_resources/INVOLVE%202012%20PublicInvolvementSystematicReviews.pdf.

[27] Page MJ, Moher D, Bossuyt PM (2021). PRISMA 2020 explanation and elaboration: updated guidance and exemplars for reporting systematic reviews. BMJ.

[28] Baldwin S, Malone M, Murrells T (2021). A mixed-methods feasibility study of an intervention to improve men’s mental health and wellbeing during their transition to fatherhood. BMC Public Health.

[29] Baral JEV, Guzman R de (2021). Anxieties and Coping among Filipino New Fathers with Postnatal Depression. J Fam Issues.

[30] Barclay L, Donovan J, Genovese A (1996). Men’s experiences during their partner’s first pregnancy: a grounded theory analysis. *Aust J Adv Nurs*.

[31] Dallos R, Nokes L (2011). Distress, Loss, and Adjustment Following the Birth of a Baby: A Qualitative Exploration of One New Father’s Experiences. J Constr Psychol.

[32] Finnbogadóttir H, Crang Svalenius E, Persson EK (2003). Expectant first-time fathers’ experiences of pregnancy. Midwifery.

[33] Hall WA (1994). New fatherhood: myths and realities. Public Health Nurs.

[34] Hodgson S, Painter J, Kilby L (2021). The Experiences of First-Time Fathers in Perinatal Services: Present but Invisible. Healthcare (Basel).

[35] Johansson M, Thomas J, Hildingsson I (2016). Swedish fathers contemplate the difficulties they face in parenthood. Sexual and Reproductive Healthcare.

[36] Johansson M, Benderix Y, Svensson I (2020). Mothers’ and fathers’ lived experiences of postpartum depression and parental stress after childbirth: a qualitative study. Int J Qual Stud Health Well-being.

[37] Kowlessar O, Fox JR, Wittkowski A (2014). First-time fathers’ experiences of parenting during the first year. Journal of Reproductive and Infant Psychology.

[38] Lagarto A, Duaso MJ (2022). Fathers’ experiences of fetal attachment: A qualitative study. Infant Ment Health J.

[39] Yuan Ling Marjorie K, Li Cheng Anna T, Shorey S (2021). Perceptions of Distressed Fathers in the Early Postpartum Period: A Descriptive Qualitative Study. J Fam Issues.

[40] Machin AJ (2015). Mind the gap: the expectation and reality of involved fatherhood. Fathering A J of Theory, Res and Pract about Men as Fathers.

[41] Nešporová O (2019). Hazy Transition to Fatherhood: The Experiences of Czech Fathers. J Fam Issues.

[42] Pålsson P, Persson EK, Ekelin M (2017). First-time fathers experiences of their prenatal preparation in relation to challenges met in the early parenthood period: Implications for early parenthood preparation. Midwifery.

[43] Shorey S, Dennis CL, Bridge S (2017). First-time fathers’ postnatal experiences and support needs: A descriptive qualitative study. J Adv Nurs.

[44] Shorey S, Ang L, Goh ECL (2019). Paternal involvement of Singaporean fathers within six months postpartum: a follow-up qualitative study. Midwifery.

[45] Wilkes L, Mannix J, Jackson D (2012). “I am going to be a dad”: experiences and expectations of adolescent and young adult expectant fathers. J Clin Nurs.

[46] Clifford‐Motopi A, Fisher I, Kildea S (2022). Hearing from First Nations Dads: Qualitative yarns informing service planning and practice in urban Australia. Fam Relat.

[47] Davenport C, Swami V (2023). “What Can I Do to Not Have This Life”? A Qualitative Study of Paternal Postnatal Depression Experiences among Fathers in the United Kingdom. Issues Ment Health Nurs.

[48] Reay M, Mayers A, Knowles-Bevis R (2023). Understanding the Barriers Fathers Face to Seeking Help for Paternal Perinatal Depression: Comparing Fathers to Men Outside the Perinatal Period. Int J Environ Res Public Health.

[49] Deave T, Johnson D (2008). The transition to parenthood: what does it mean for fathers?. J Adv Nurs.

[50] Fägerskiöld A (2008). A change in life as experienced by first-time fathers. Scand J Caring Sci.

[51] Ngai FW, Lam W (2020). The experience of first‐time Hong Kong Chinese fatherhood: a qualitative exploratory study. Nursing and Health Sciences.

[52] John WS, Cameron C, McVeigh C (2005). Meeting the challenge of new fatherhood during the early weeks. J Obstet Gynecol Neonatal Nurs.

[53] Rominov H, Giallo R, Pilkington PD (2018). “Getting help for yourself is a way of helping your baby:” fathers’ experiences of support for mental health and parenting in the perinatal period. Psychology of Men and Masculinity.

[54] Fletcher R, StGeorge J, Newman L (2020). Male callers to an Australian perinatal depression and anxiety help line-Understanding issues and concerns. Infant Ment Health J.

[55] Fenwick J, Bayes S, Johansson M (2012). A qualitative investigation into the pregnancy experiences and childbirth expectations of Australian fathers-to-be. Sexual and Reproductive Healthcare.

[56] Gottfredsdóttir H (2005). Prospective first-time fathers and their views on fatherhood in the context of new policy on parental leave in Iceland. Birth Issues.

[57] Pedersen SC, Maindal HT, Ryom K (2021). “I Wanted to Be There as a Father, but I Couldn’t”: A Qualitative Study of Fathers’ Experiences of Postpartum Depression and Their Help-Seeking Behavior. Am J Mens Health.

[58] Shorey S, Ang L, Goh ECL (2018). Lived experiences of Asian fathers during the early postpartum period: Insights from qualitative inquiry. Midwifery.

[59] Golian Tehrani S, Bazzazian S, Dehghan Nayeri N (2015). Pregnancy Experiences of First-Time Fathers in Iran: A Qualitative Interview Study. Iran Red Crescent Med J.

[60] Rayburn SR, Coatsworth JD, MacPhee D (2021). Becoming Fathers: A Mixed-methods Study of the Feasibility and Acceptability of a Mindfulness-Based Group Intervention for Perinatal Fathers. J Child Fam Stud.

[61] Kaner A, Cwikel J, Segal-Engelchin D (2024). The transition to fatherhood – evaluation of an online intervention for new fathers. Psychol Health Med.

[62] Lévesque S, Bisson V, Fernet M (2021). A study of the transition to parenthood: new parents’ perspectives on their sexual intimacy during the perinatal period. Sex Relation Ther.

[63] Davenport C, Lambie J, Owen C (2022). Fathers’ experience of depression during the perinatal period: a qualitative systematic review. *JBI Evid Synth*.

[64] Bradley R, Slade P (2011). A review of mental health problems in fathers following the birth of a child. J Reprod Infant Psychol.

[65] Henwood K, Procter J (2003). The “good father”: reading men’s accounts of paternal involvement during the transition to first-time fatherhood. Br J Soc Psychol.

[66] Allen SM, Hawkins AJ (1999). Maternal Gatekeeping: Mothers’ Beliefs and Behaviors That Inhibit Greater Father Involvement in Family Work. J Marriage Fam.

[67] Thomas CR, Holmes EK (2020). Are father depression and masculinity associated with father perceptions of maternal gatekeeping?. J Fam Psychol.

[68] Pinho M, Gaunt R, Gross H (2021). Caregiving dads, breadwinning mums: pathways to the division of family roles among role-reversed and traditional parents. Marriage and Family Review.

[69] Lamb ME (2000). The history of research on father involvement. Marriage and Family Review.

[70] Polomeno V (1999). Perinatal education and grandparenting: creating an interdependent family environment. Part I: documenting the need. J Perinat Educ.

[71] Yeung W-JJ (2013). Asian Fatherhood. J Fam Issues.

[72] Hodgson S, Painter J, Kilby L The Experiences of First-Time Fathers in Perinatal Services: Present but Invisible. Healthcare (Basel).

[73] Meulen BD (2019). Mother and child are doing fine(master’s thesis). https://studenttheses.uu.nl/handle/20.500.12932/34294.

[74] Lee JY, Knauer HA, Lee SJ (2018). Father-Inclusive Perinatal Parent Education Programs: A Systematic Review. Pediatrics.

[75] Davenport C, Swami V (2023). Health visitors’ experiences of supporting fathers with paternal postnatal depression. J Health Visiting.

[76] Cottingham MD (2017). Caring moments and their men: masculine emotion practice in nursing. *NORMA*.

[77] Nash M (2018). “It’s just good to get a bit of man-talk out in the open”: men’s experiences of father-only antenatal preparation classes in Tasmania, Australia. Psychology of Men and Masculinity.

