# Supplementary material for: Exploration of fathers’ mental health and well-being concerns during the transition to fatherhood, and paternal perinatal support: scoping review
Source: BMJ Open. 2024 Nov 12;14(11):e078386. doi: 10.1136/bmjopen-2023-078386 (PMC11574476; doi:10.1136/bmjopen-2023-078386)
Supplement: online supplemental file 1 [file bmjopen-14-11-s001.pdf]

Additional File 1: Full search strategy

OVID (Medline)

Fathers in Perinatal Period
1. Father$.ab,ti.
2. Paternal.ab,ti.
3. Dad$.ab,ti.
4. 1 of 2 or 3

5. Exp fathers/
6. 4 or 5

7. Perinatal.ab,ti.
8. Postpartum.ab,ti.
9. Antenatal.ab,ti.
10. Prenatal.ab,ti.
11. Postnatal.ab,ti
12. Fatherhood.ab,ti
13. 7 or 8 or 9 or 10 or 11 or 12

14. Exp Postpartum Period/
15. 13 or 14

Mental Health and Well-being
16. Mental health.ab,ti.
17. Psychological distress.ab,ti.
18. Stress.ab,ti.
19. Anxiety.ab,ti.
20. Depression.ab,ti
21. Postpartum depression.ab,ti
22. Paternal stress.ab,ti.
23. Mental health disorder.ab,ti
24. Wellbeing.ab,ti
25. Trauma.ab,ti
26. 16 or 17 or 18 or 19 or 20 or 21 or 22 or 23 or 24 or 25

27. Exp mental health/
28. Exp Psychological distress/
29. Exp Anxiety/
30. Exp Depression/
31. Exp Depression, Postpartum/
32. Exp Mental disorders/
33. 27 or 28 or 29 or 30 or 31 or 32
34. 26 or 33

Qualitative Research
35. Qualitative.ab,ti.
36. Interview$.ab,ti
37. Mixed method$.ab,ti

38. Focus group$.ab,ti.
39. Case stud$.ab,ti
40. Experience$.ab,ti.
41. Exploratory.ab,ti.
42. Descriptive$.ab,ti.
43. Thematic analysis.ab,ti
44. Observation.ab,ti
45. View$.ab,ti.
46. Belief$.ab,ti
47. Perception$.ab,ti
48. 35 or 36 or 37 or 38 or 39 or 40 or 41 or 42 or 43 or 44 or 45 or 46 or 47

49. Exp qualitative Research/
50. Exp focus groups/
51. Exp Observation/
52. 49 or 50 or 51
53. 48 or 52
54. 6 and 15 and 34 and 53

Accessing Support and Interventions
55. Support.ab,ti.
56. Accessing support.ab,ti.
57. Support need$.ab,ti.
58. Barrier$.ab,ti.
59. Facilitator$.ab,ti.
60. Mental health service$.ab,ti.
61. Intervention$.ab,ti.
62. Therap$.ab,ti.
63. Treatment$.ab,ti.
64. Program$.ab,ti.
65. Preventative.ab,ti
66. 55 or 56 or 57 or 58 or 59 or 60 or 61 or 62 or 63 or 64 or 65

67. Exp Mental Health Services/
68. 66 or 67
69. 54 and 68 = 423 papers

Found 8/12 tracer papers, 4 not in database

OVID (Embase)

Fathers in Perinatal Period
1. Father$.ab,ti
2. Dad$.ab,ti
3. Paternal.ab,ti
4. 1 or 2 or 3

5. Exp father/
6. 4 or 5

7. Perinatal.ab,ti
8. Postpartum.ab,ti
9. Antenatal.ab,ti
10. Prenatal.ab,ti
11. Postnatal.ab,ti
12. Fatherhood.ab,ti
13. 7 or 8 or 9 or 10 or 11 or 12

14. Exp perinatal period/
15. Exp prenatal period/
16. 14 or 15
17. 13 or 16

Mental Health and Well-being

18. Mental health.ab,ti
19. Psychological distress.ab,ti
20. Stress.ab,ti
21. Anxiety.ab,ti
22. Depression.ab,ti
23. Postpartum depression.ab,ti
24. Paternal stress.ab,ti
25. Wellbeing.ab,ti
26. Mental disorder.ab,ti
27. 18 or 19 or 20 or 21 or 22 or 23 or 24 or 25 or 26

28. Exp mental health/
29. Exp anxiety/
30. Exp depression/
31. Exp postnatal depression/
32. Exp paternal stress/
33. Exp wellbeing/
34. 28 or 29 or 30 or 31 or 32 or 33
35. 27 or 34

Qualitative Research

36. Qualitative.ab,ti
37. Interview$.ab,ti
38. Mixed method$.ab,ti
39. Focus group$.ab,ti
40. Case stud$.ab,ti
41. Experience$.ab,ti
42. Exploratory.ab,ti
43. Descriptive.ab,ti
44. View$.ab,ti
45. Perception$.ab,ti
46. Belief$.ab,ti
47. Thematic analysis.ab,ti
48. Observation.ab,ti
49. 36 or 37 or 38 or 39 or 40 or 41 or 42 or 43 or 44 or 45 or 46 or 47 or 48

50. Exp qualitative research/
51. Exp semi-structured interview/
52. Exp thematic analysis/
53. Exp observation/
54. 50 or 51 or 52 or 53 or 54 or 55
55. 49 or 54
56. 6 and 17 and 35 and 55

Accessing Support and Interventions
57. Support.ab,ti
58. Accessing support.ab,ti
59. Support needs.ab,ti
60. Barrier$.ab,ti
61. Facilitator$.ab,ti
62. Mental health service$.ab,ti
63. Intervention$.ab,ti
64. Therap$.ab,ti
65. Treatment$.ab,ti
66. Program$.ab,ti
67. Preventative.ab,ti
68. 57 or 58 or 59 or 60 or 61 or 62 or 63 or 64 or 65 or 66 or 67
69. Exp mental health service/
70. 68 or 69
71. 56 and 70

Found 8/12 tracer papers, 4 not in database

OVID (PsycINFO)

Fathers in Perinatal Period
1. Father$.ab,ti
2. Dad$.ab,ti
3. Paternal.ab,ti
4. 1 or 2 or 3

5. Exp fathers/
6. 4 or 5

7. Perinatal.ab,ti
8. Postpartum.ab,ti
9. Antenatal.ab,ti
10. Prenatal.ab,ti
11. Postnatal.ab,ti
12. Fatherhood.ab,ti
13. 7 or 8 or 9 or 10 or 11 or 12

14. Exp perinatal period/
15. 13 or 14

Mental Health and Well-being

16. Mental health.ab,ti
17. Psychological distress.ab,ti
18. Stress.ab,ti
19. Anxiety.ab,ti
20. Depression.ab,ti
21. Postpartum depression.ab,ti
22. Paternal stress.ab,ti
23. Wellbeing.ab,ti
24. Mental disorder.ab,ti
25. 16 or 17 or 18 or 19 or 20 or 21 or 22 or 23 or 24

26. Exp mental health/
27. Exp stress/
28. Exp psychological stress/
29. Exp anxiety/
30. Exp postpartum depression/
31. Exp wellbeing/
32. Exp mental disorders/
33. 26 or 27 or 28 or 29 or 30 or 31 or 32
34. 25 or 33

Qualitative Research

35. Qualitative.ab,ti
36. Interview$.ab,ti
37. Mixed method$.ab,ti
38. Focus group$.ab,ti
39. Case stud$.ab,ti
40. Experience$.ab,ti
41. Exploratory.ab,ti
42. Descriptive.ab,ti
43. Thematic analysis.ab,ti
44. Observation$.ab,ti
45. View$.ab,ti
46. Perception$.ab,ti
47. Belief$.ab,ti
48. 35 or 36 or 37 or 38 or 39 or 40 or 41 or 42 or 43 or 44 or 45 or 46 or 47

49. Exp qualitative methods/
50. Exp thematic analysis/
51. Exp mixed methods research/
52. Exp interviews/
53. Exp focus groups/
54. 49 or 50 or 51 or 52 or 53
55. 48 or 54
56. 6 and 15 and 34 and 55

Accessing Support and Interventions

57. Support.ab,ti

58. Psychological support.ab,ti
59. Accessing support.ab,ti
60. Support needs.ab,ti
61. Barrier$.ab,ti
62. Facilitator$.ab,ti
63. Mental health service$.ab,ti
64. Intervention$.ab,ti
65. Therap$.ab,ti
66. Treatment$.ab,ti
67. Program$.ab,ti
68. Preventative.ab,ti
69. 57 or 58 or 59 or 60 or 61 or 62 or 63 or 64 or 65 or 66 or 67 or 68

70. Exp mental health services/
71. Exp intervention/
72. Exp treatment/
73. Exp mental health programs/
74. 70 or 71 or 72 or 73
75. 69 or 74
76. 56 and 75

Found 7/12 tracer papers, 5 not in database

EBSCOhost (Cumulative Index to Nursing and Allied Health Literature CINAHL)

Fathers in Perinatal Period
1. TI (father*) OR AB (father*)
2. TI (paternal) OR AB (paternal)
3. TI (dad*) OR AB (dad*)
4. S1 OR S2 OR S3

5. (MH "Fathers+")
6. S4 OR S5

7. TI (perinatal) OR AB (perinatal)
8. TI (postnatal) OR AB (postnatal)
9. TI (prenatal) OR AB (prenatal)
10. TI (postpartum) OR AB (postpartum)
11. TI (antenatal) OR AB (antenatal)
12. TI (fatherhood) OR AB (fatherhood)
13. S7 OR S8 OR S9 OR S10 OR S11 OR S12

14. (MH "Postnatal Period+")
15. (MH "Fatherhood")
16. S14 OR S15
17. S13 OR S16

Mental Health and Well-being

18. TI (“mental health”) OR AB (“mental health”)
19. TI (“psychological distress”) OR AB (“psychological distress”)
20. TI (stress) OR AB (stress)
21. TI (anxiety) OR AB (anxiety)
22. TI (depression) OR AB (depression)
23. TI (“postpartum depression”) OR AB (“postpartum depression”)
24. TI (“paternal stress”) OR AB (“paternal stress”)
25. TI (wellbeing) OR AB (wellbeing)
26. TI (“mental disorder”) OR AB (“mental disorder”)
27. S18 OR S19 OR S20 OR S21 OR S22 OR S23 OR S24 OR S25 OR S26

28. (MH “mental health”)
29. (MH “psychological distress”)
30. (MH “stress+”)
31. (MH “anxiety+”)
32. (MH “depression+”)
33. (MH “depression, postpartum”)
34. (MH “psychological wellbeing”)
35. (MH “mental disorders+”)
36. S28 OR S29 OR S30 OR S31 OR S32 OR S33 OR S34 OR S35
37. S27 OR S36

Qualitative Research

38. TI (qualitative) OR AB (qualitative)
39. TI (interview*) OR AB (interview*)
40. TI (“mixed method*” OR AB (“mixed method*”)
41. TI (“focus group*”) OR AB (“focus group*”)
42. TI (“case stud*”) OR AB (“case stud*”)
43. TI (experience*) OR AB (experience*)
44. TI (exploratory) OR AB (exploratory)
45. TI (descriptive) OR AB (descriptive)
46. TI (“thematic analysis”) OR AB (“thematic analysis”)
47. TI (observation) OR AB (observation)
48. TI (view*) OR AB (view*)
49. TI (perception*) OR AB (perception*)
50. TI (belief*) OR AB (belief*)
51. S38 OR S39 OR S40 OR S41 OR S42 OR S43 OR S44 OR S45 OR S46 OR S47 OR S48 OR S49 OR S50 OR S51

52. (MH “Qualitative studies+”)
53. (MH “semi-structured interview”)
54. (MH “focus groups”)
55. (MH “case studies”)
56. (MH “thematic analysis”)
57. S52 OR S53 OR S54 OR S55 OR S56
58. S51 OR S57
59. S6 AND S17 AND S37 AND S58

Accessing Support and Interventions

60. TI (support) OR AB (support)

61. TI (“psychological wellbeing”) OR AB (“psychological wellbeing”)
62. TI (“accessing support”) OR AB (“accessing support”)
63. TI (“support needs*”) OR AB (“support need*”)
64. TI (barrier*) OR AB (barrier*)
65. TI (facilitator*) OR AB (facilitator*)
66. TI (“mental health service*”) OR AB (mental health service*”)
67. TI (intervention*) OR AB (intervention*)
68. TI (therap*) OR AB (therap*)
69. TI (treatment*) OR AB (treatment*)
70. TI (program*) OR AB (program*)
71. TI (preventative) OR AB (preventative)
72. S60 OR 61 OR S62 OR S63 OR S64 OR S65 OR S66 OR S67 OR S68 OR S69 OR S70 OR S71

73. (MH “support, psychological+”)
74. (MH “mental health services+”)
75. (MH “psychological intervention+”)
76. S73 OR S74 OR S75
77. S72 OR 76
78. S59 AND S77

Found 7/12 tracer papers, 5 not in database

Scopus

TITLE-ABS-KEY (father* OR paternal OR dad*)
AND

TITLE-ABS-KEY (perinatal OR postpartum OR antenatal OR postnatal OR prenatal OR fatherhood)
AND

TITLE-ABS-KEY ("mental health" OR depression OR anxiety OR "postnatal depression" OR "psychological distress" OR wellbeing OR stress OR "mental disorder")
AND

TITLE-ABS-KEY (qualitative OR "thematic analysis" OR interview* OR "focus group" OR exploratory OR descriptive OR "case stud*" OR "Mixed method*" OR observation)
AND

TITLE-ABS-KEY (support OR "accessing support" OR "support need*" OR "mental health service*" OR intervention* OR therap* OR treatment* OR program* OR barrier* OR facilitator*)

Found 11/12 papers, 1 not in database

ProQuest (Sociology Collection: Applied Social Sciences Index and Abstracts ASSIA)

Fathers in Perinatal Period

1. ti(father*) OR ab(father*)
2. ti(dad*) OR ab(dad*)
3. ti(paternal) OR ab(paternal)
4. 1 OR 2 OR 3

5. MAINSUBJECT.EXACT.EXPLODE("Fathers")
6. 4 OR 5

7. ti(perinatal) OR ab(perinatal)
8. ti(postnatal) OR ab(postnatal)
9. ti(prenatal) OR ab(prenatal)
10. ti(antenatal) OR ab(antenatal)
11. ti(postpartum) OR ab(postpartum)
12. ti(Fatherhood) OR ab(Fatherhood)
13. 7 OR 8 OR 9 OR 10 OR 11 OR 12

14. MAINSUBJECT.EXACT.EXPLODE("Perinatal period")
15. MAINSUBJECT.EXACT. EXPLODE("Antenatal")
16. MAINSUBJECT.EXACT.EXPLODE("Fatherhood")
17. 14 OR 15 OR 16
18. 13 OR 17

Mental Health and Well-being

19. ti("mental health") OR ab("mental health")
20. ti("psychological distress") OR ab("psychological distress")
21. ti(stress) OR ab(stress)
22. ti(anxiety) OR ab (anxiety)
23. ti(depression) OR ab(depression)
24. ti("postpartum depression") OR ab("postpartum depression")
25. ti("paternal stress") OR ab("paternal stress")
26. ti(wellbeing) OR ab(wellbeing)
27. ti("mental disorder") OR ab("mental disorder")
28. 19 OR 20 OR 21 OR 22 OR 23 OR 24 OR 25 OR 26 OR 27

29. MAINSUBJECT.EXACT.EXPLODE("Mental health")
30. MAINSUBJECT.EXACT.EXPLODE(“Psychological Distress”)
31. MAINSUBJECT.EXACT.EXPLODE("Stress")
32. MAINSUBJECT.EXACT.EXPLODE("Anxiety")
33. MAINSUBJECT.EXACT.EXPLODE("Depression")
34. MAINSUBJECT.EXACT.EXPLODE("Postnatal depression")
35. MAINSUBJECT.EXACT.EXPLODE("Psychological wellbeing")
36. 29 OR 30 OR 31 OR 32 OR 33 OR 34 OR 35
37. 28 OR 36

Qualitative Research

38. ti(qualitative) OR ab(qualitative)
39. ti(interview*) OR ab(interview*)
40. ti("mixed method*") OR ab("mixed method*")
41. ti(“focus group*”) OR ab(“focus group*”)

42. ti("case stud*") OR ab("case stud*")
43. ti(experience*) OR ab(experience*)
44. ti(exploratory) OR ab(exploratory)
45. ti(descriptive) OR ab(descriptive)
46. ti("thematic analysis") OR ab("thematic analysis")
47. ti(observation) OR ab(observation)
48. ti(view*) OR ab(view*)
49. ti(perception*) OR ab(perception*)
50. ti(belief*) OR ab(belief*)
51. 38 OR 39 OR 40 OR 41 OR 42 OR 43 OR 44 OR 45 OR 46 OR 47 OR 48 OR 49 OR 50

52. MAINSUBJECT.EXACT.EXPLODE("Qualitative research")
53. MAINSUBJECT.EXACT.EXPLODE("Interviews")
54. MAINSUBJECT.EXACT.EXPLODE("Focus groups")
55. MAINSUBJECT.EXACT.EXPLODE("Case studies")
56. MAINSUBJECT.EXACT.EXPLODE("Observation")
57. 52 OR 53 OR 54 OR 55 OR 56
58. 51 OR 57
59. 6 AND 18 AND 37 AND 58

Accessing Support and Interventions
60. ti(support) OR ab(support)
61. ti("psychological distress") OR ab("psychological distress")
62. ti("accessing support") OR ab("accessing support")
63. ti("support need*") OR ab("support need*")
64. ti(barrier*) OR ab(barrier*)
65. ti(facilitator*) OR ab(facilitator*)
66. ti("mental health service*") OR ab("mental health service*")
67. ti(intervention*) OR ab(intervention*)
68. ti(therap*) OR ab(therap*)
69. ti(treatment*) OR ab(treatment*)
70. ti(program*) OR ab(program*)
71. ti(preventative) OR ab(preventative)
72. 60 OR 61 OR 62 OR 63 OR 64 OR 65 OR 66 OR 67 OR 68 OR 69 OR 70 OR 71

73. MAINSUBJECT.EXACT.EXPLODE("Psychological distress")
74. MAINSUBJECT.EXACT.EXPLODE("Barriers")
75. MAINSUBJECT.EXACT.EXPLODE("Facilitators")
76. MAINSUBJECT.EXACT.EXPLODE("Mental health services")
77. MAINSUBJECT.EXACT.EXPLODE("Intervention")
78. MAINSUBJECT.EXACT.EXPLODE("Treatment")
79. MAINSUBJECT.EXACT.EXPLODE("Programmes")
80. MAINSUBJECT.EXACT.EXPLODE("Prevention")
81. 73 OR 74 OR 75 OR 76 OR 77 OR 78 OR 79 OR 80
82. 72 OR 81
59 AND 82

Found 3/12 papers, 9 not in database

Open Access and Theses Dissertations

Fathers in Perinatal Period

1. (title:(fathers)) OR (abstract:(fathers))
2. (title:(dads)) OR (abstract:(dads))
3. (title:(paternal)) OR (abstract:(paternal))
4. 1 OR 2 OR 3

5. (title:(perinatal)) OR (abstract:(perinatal))
6. title:(postnatal)) OR ((abstract:(postnatal))
7. (title:(antenatal)) OR (abstract:(antenatal))
8. (title:(prenatal)) OR (abstract:(prenatal))
9. (title:(postpartum)) OR (abstract:(postpartum))
10. (title:(fatherhood)) OR (abstract:(fatherhood))
11. 5 OR 6 OR 7 OR 8 OR 9 OR 10

Mental Health and Well-being

12. (abstract:(wellbeing)) OR (title:(wellbeing))
13. (abstract:(depression)) OR (title:(depression))
14. (abstract:(anxiety)) OR (title:(anxiety))
15. (abstract:(stress)) OR (title:(stress))
16. (abstract:(mental health)) OR (title:(mental health))
17. (title:(postnatal AND depression)) OR (abstract:(postnatal AND depression))
18. (abstract:(psychological AND distress)) OR (title:(psychological AND distress))
19. (title:(paternal AND stress)) OR (abstract:(paternal AND stress))
20. (abstract:(mental AND disorder)) OR (title:(mental AND disorder))
21. 12 OR 13 OR 14 OR 15 OR 16 OR 17 OR 18 OR 19 OR 20

Qualitative Research

22. (title:(qualitative)) OR (abstract:(qualitative))
23. (title:(interview)) OR (abstract:(interview))
24. (title:(mixed AND method)) OR (abstract:(mixed AND method))
25. (title:(focus AND group))OR (abstract:(focus AND group))
26. (title:(case AND study))OR (abstract:(focus AND group))
27. (abstract:(experience)) OR (title:(experience))
28. (title:(exploratory)) OR (abstract:(exploratory))
29. (title:(observation)) OR (abstract:(observation))
30. (abstract:(perception)) OR (title:(perception))
31. (title:(belief)) OR (abstract:(belief))
32. 22 OR 23 OR 24 OR 25 OR 26 OR 27 OR 28 OR 29 OR 30 OR 31

32. 4 AND 11 AND 21 AND 32

Accessing Support and Interventions

33. (title:(barrier)) OR (abstract:(barrier))
34. (title:(facilitator)) OR (abstract:(facilitator))
35. (title:(intervention)) OR (abstract:(intervention))
36. (title:(treatment)) OR (abstract:(treatment))
37. (title:(therapy)) OR (abstract:(therapy))
38. (title:(programme)) OR (abstract:(programme))
39. (title:(preventative)) OR (abstract:(preventative))

40. (title:(accessing support)) OR abstract:(accessing AND support))
41. (abstract:(psychological AND distress)) OR (title:(psychological AND distress)
42. 33 OR 34 OR 35 OR 36 OR 37 OR 38 OR 39 OR 40 OR 41 OR 42
43. 32 AND 42
