# Supplementary material for: Exploration of fathers’ mental health and well-being concerns during the transition to fatherhood, and paternal perinatal support: scoping review
Source: BMJ Open. 2024 Nov 12;14(11):e078386. doi: 10.1136/bmjopen-2023-078386 (PMC11574476; doi:10.1136/bmjopen-2023-078386)
Supplement: online supplemental file 2 [file bmjopen-14-11-s002.pdf]

Additional File 2. JBI Qualitative Research Critical Appraisal Table

First Author (Date)	1.	2.	3.	4.	5.	6.	7.	8.	9.	10.	Overall Appraisal	Comments
Baldwin (2019)	No	Yes	Yes	Yes	Yes	No	Yes	Yes	Yes	Yes	Include	• There is no philosophical orientation on which the study is based, just states qualitative methodology, can be considered less rigorous in design.<br>• Not aware of the researchers cultural and theoretical orientation.
Baldwin (2021)	No	Yes	Yes	Yes	Yes	No	No	Yes	Yes	Yes	Include	• There is no philosophical/theoretical orientation on which the study is based, just states qualitative aspect within a mixed method observational cohort design, can be considered less rigorous in design for the qualitative part of study.<br>• Qualitative approach is not specifically stated (theoretical or philosophical orientation). However, thematic analysis is considered an appropriate type of analysis for more descriptive/less interpretive qualitative approaches, which is shown in this study exploring satisfaction of an intervention and has been employed effectively.<br>• Not aware of the researchers cultural and theoretical orientation.<br>• Researcher interpretation is not acknowledged. No reflection of role or response to events throughout study.
Baral (2021)	No	Yes	Yes	Yes	Yes	No	No	Yes	Yes	Yes	Include	• There is no philosophical/theoretical orientation on which the study is based, just states qualitative methodology, can be considered less rigorous in design.<br>• Not aware of the researchers cultural and theoretical orientation.<br>• Researcher interpretation is not acknowledged. No reflection of role or response to events throughout study.
Barclay (1996)	Yes	Yes	Yes	Yes	Yes	No	No	Yes	No	Yes	Include	• Not aware of the researchers cultural and theoretical orientation.<br>• Researcher interpretation is not acknowledged. No reflection of role or response to events throughout study.<br>• No statement on the ethical approval or body.

Clifford-Montop (2022)	No	Yes	Yes	Yes	Yes	No	No	Yes	Yes	Yes	Include	• There is no philosophical/theoretical orientation on which the study is based, just states qualitative methodology, can be considered less rigorous in design.<br>• Beliefs and values are not declared in this report.<br>• Influence of the researcher is not addressed particularly influence on data collection.
Dallos (2011)	Yes	Yes	Yes	Yes	Yes	No	Yes	Yes	No	Yes	Include	• Not aware of the researchers cultural and theoretical orientation.<br>• No ethical statement or body acknowledged.
Darwin (2017)	Yes	Yes	Yes	Yes	Yes	Yes	No	Yes	Yes	Yes	Include	• Beliefs and values are declared in this report.<br>• Influence of the researcher is not addressed particularly influence on data collection.
Davenport (2023)	Yes	Yes	Yes	Yes	Yes	No	Yes	Yes	Yes	Yes	Include	• Would be good to be aware of more of the researchers' beliefs and values and their potential influence on the study declared.
Deave (2008)	No	Yes	Yes	Yes	Yes	No	No	Yes	Yes	Yes	Include	• There is no philosophical orientation on which the study is based, just states cross sectional study and qualitative, can be considered lees rigorous in design.<br>• No initial methodological position is stated however, thematic analysis a mainly descriptive approach has been employed effectively, presuming the study has a more descriptive rather than interpretive stance.<br>• Not aware of the researchers cultural and theoretical orientation.<br>• Researcher interpretation is not acknowledged. No reflection of role or response to events throughout study.
Edhborg (2016)	Yes	Yes	Yes	Yes	Yes	No	No	Yes	Yes	Yes	Include	• Not aware of the researchers cultural and theoretical orientation.<br>• Researcher interpretation is not acknowledged. No reflection of role or response to events throughout study.<br>• Suitable to use content analysis with descriptive qualitative approaches, employing a relatively low level of interpretation.
Fagerskiold (2008)	Yes	Yes	Yes	Yes	Yes	No	No	Yes	Yes	Yes	Include	• Not aware of the researchers cultural and theoretical orientation.

												• Researcher interpretation is not acknowledged. No reflection of role or response to events throughout study.
Fei-Wan (2019)	Yes	Yes	Yes	Yes	Yes	No	Yes	Yes	Yes	Yes	Include	• Maintained a reflexive diary to reflect on their thoughts, feelings, and observations within data analysis.<br>• Not aware of the researchers cultural and theoretical orientation.
Fenwick 2012)	Yes	Yes	Yes	Yes	Yes	No	No	Yes	Yes	Yes	Include	• Not aware of the researchers cultural and theoretical orientation.<br>• Researcher interpretation is not acknowledged. No reflection of role or response to events throughout study.
Finnbogadottir (2002)	Yes	Yes	Yes	Yes	Yes	No	No	Yes	Yes	Yes	Include	• Not aware of the researchers cultural and theoretical orientation.<br>• Researcher interpretation is not acknowledged. No reflection of role or response to events throughout study.
Fletcher (2019)	No	Yes	Yes	Yes	Yes	No	No	Yes	Yes	Yes	Include	• There is no philosophical orientation on which the study is based, or specific stated methodology, presumed qualitative as thematic analysis has been employed to analyse data qualitatively.<br>• Counselling narrative phone calls are considered a qualitative method; however, interviews may be considered more effective in exploring phenomena of interest.<br>• Qualitative approach is not specifically stated (theoretical or philosophical orientation). However, thematic analysis is considered an appropriate type of analysis for more descriptive/less interpretive qualitative approaches, which is shown in this study and has been employed effectively.<br>• Not aware of the researchers cultural and theoretical orientation.<br>• Researcher interpretation is not acknowledged. No reflection of role or response to events throughout study.
Gottfredsdottir (2005)	No	Yes	Yes	Yes	Yes	No	No	Yes	Yes	Yes	Include	• There is no philosophical orientation on which the study is based, just states qualitative aspect within a mixed method design, can be considered less rigorous in design for the qualitative part of study.<br>• Qualitative approach is not specifically stated. However, thematic content analysis is considered an appropriate type of

												analysis for more descriptive/less interpretive qualitative approaches, which is shown in this study describing their views and educational needs within fatherhood.<br>• Not aware of the researchers cultural and theoretical orientation.<br>• Researcher interpretation is not acknowledged. No reflection of role or response to events throughout study.
Hall (1994)	Yes	Yes	Yes	Yes	Yes	No	No	Yes	No	Yes	Include	• Not aware of the researchers cultural and theoretical orientation.<br>• Researcher interpretation is not acknowledged. No reflection of role or response to events throughout study.<br>• No ethical statement or body acknowledged.
Hodgson, (2021)	Yes	Yes	Yes	Yes	Yes	No	No	Yes	Yes	Yes	Include	• Not aware of the researchers cultural and theoretical orientation.<br>• Researcher interpretation is not acknowledged. No reflection of role or response to events throughout study.
Johannsson (2016)	No	Yes	Yes	Yes	Yes	No	No	Yes	Yes	Yes	Include	• There is no philosophical orientation on which the study is based, just states qualitative aspect within a mixed method design, can be considered less rigorous in design for the qualitative part of study.<br>• Study employs open ended questions as means of collecting qualitative data, not as rigour as interviews, but can still be considered appropriate in collecting qualitative data with an approach that is not demanding of high levels of interpretation, such as a phenomenological approach.<br>• Qualitative approach is not specifically stated (theoretical or philosophical orientation). However, content analysis is considered an appropriate type of analysis for more descriptive/less interpretive qualitative approaches, which is shown in this study and has been employed effectively.<br>• Not aware of the researchers cultural and theoretical orientation.
Johansson, (2020)	Yes	Yes	Yes	Yes	Yes	No	No	Yes	Yes	Yes	Include	• Not aware of the researchers cultural and theoretical orientation.<br>• Researcher interpretation is not acknowledged. No reflection of role or response to events throughout study.
Kaner (2023)	No	Yes	Yes	Yes	Yes	No	No	Yes	Yes	Yes	Include	• There is no philosophical orientation on which the study is based, just states qualitative aspect within a mixed method

												design, can be considered less rigorous in design for the qualitative part of study.<br>• Not aware of the researchers cultural and theoretical orientation.<br>• Researcher interpretation is not acknowledged. No reflection of role or response to events throughout study.
Kowlessar (2014)	Yes	Yes	Yes	No	Yes	No	No	Yes	Yes	Yes	Include	• Analysis only focuses on meanings that all participants/majority address and does not capture individual participants reported meanings, that may show contrast in viewpoints. This is not representative of a phenomenological approach.<br>• Not aware of the researchers cultural and theoretical orientation.<br>• Researcher interpretation is not acknowledged. No reflection of role or response to events throughout study.
Lagarto (2021)	Yes	Yes	Yes	Yes	Yes	No	No	Yes	No	Yes	Include	• Not aware of the researchers cultural and theoretical orientation.<br>• Researcher interpretation is not acknowledged. Slight reflection of role but only one comment regarding how inexperience may impact analysis.<br>• No statement on the ethical approval or body.
Ling (2021)	Yes	Yes	Yes	Yes	Yes	No	Yes	Yes	Yes	Yes	Include	• Not aware of the researchers cultural and theoretical orientation.
Machin (2015)	No	Yes	Yes	No	Yes	No	No	Yes	No	Yes	Include	• There is no philosophical orientation on which the study is based, just states qualitative aspect within a mixed method design, can be considered less rigorous in design for the qualitative part of study.<br>• Unknown type of analysis, unable to state whether analysis is congruent with methodological approach as restricted to just a qualitative approach.<br>• Not aware of the researchers cultural and theoretical orientation.<br>• No statement on the ethical approval or body.
Nesporova (2019)	Yes	Yes	Yes	Yes	Yes	No	No	Yes	No	Yes	Include	• Not aware of the researchers cultural and theoretical orientation.<br>• Researcher interpretation is not acknowledged. No reflection of role or response to events throughout study.

												• No ethical statement or body acknowledged.
Pallson (2017)	Yes	Yes	Yes	No	Yes	No	No	Yes	Yes	Yes	Include	• Analysis only focuses on meanings that all participants address and does not capture individual participants reported meanings that may show contrast in viewpoints. This is not representative of a phenomenological approach.<br>• Not aware of the researchers cultural and theoretical orientation.<br>• Researcher interpretation is not acknowledged. No reflection of role or response to events throughout study.
Pedersen, (2021)	Yes	Yes	Yes	Yes	Yes	No	No	Yes	Yes	Yes	Include	• Not aware of the researchers cultural and theoretical orientation.<br>• Researcher interpretation is not acknowledged. No reflection of role or response to events throughout study.
Rayburn, (2021)	No	Yes	Yes	Yes	Yes	No	No	Yes	Yes	Yes	Include	• There is no philosophical orientation on which the study is based, just states qualitative aspect within a mixed method design, can be considered less rigorous in design for the qualitative part of study.<br>• Used open ended question data from surveys, not considered best means of data collection, however phenomena of interest was to assess satisfaction of sessions, suited to this method of data collection.<br>• Qualitative approach is not specifically stated (theoretical or philosophical orientation). However, thematic analysis is considered an appropriate type of analysis for more descriptive/less interpretive qualitative approaches, which is shown in this study exploring satisfaction of an intervention and has been employed effectively.
Reay (2023)	Yes	Yes	Yes	Yes	Yes	No	Yes	Yes	Yes	Yes	Include	• Maintained a reflexive diary to reflect on their thoughts, feelings, and observations within data analysis.<br>• Not aware of the researchers cultural and theoretical orientation.
Rominov (2018)	Yes	Yes	Yes	Yes	Yes	No	No	Yes	Yes	Yes	Include	• Not aware of the researchers cultural and theoretical orientation.<br>• Researcher interpretation is not acknowledged. No reflection of role or response to events throughout study.

Shorey (2017)	Yes	Yes	Yes	Yes	Yes	No	No	Yes	Yes	Yes	Include	• Not aware of the researchers cultural and theoretical orientation.<br>• Researcher interpretation is not acknowledged. No reflection of role or response to events throughout study. No explanation of relationship between researcher and participants.
Shorey (2018)	Yes	Yes	Yes	Yes	Yes	No	No	Yes	Yes	Yes	Include	• Not aware of the researchers cultural and theoretical orientation.<br>• Researcher interpretation is not acknowledged. No reflection of role or response to events throughout study.
Shorey (2019)	Yes	Yes	Yes	Yes	Yes	No	Yes	Yes	Yes	Yes	Include	• Not aware of the researchers cultural and theoretical orientation.<br>• Researcher examines her role and influence of gender differences within data collection. Researcher also reports how they respond to any concerns by encouraging participants to talk without hesitation.
St. John, (2005)	Yes	Yes	Yes	Yes	Yes	No	No	Yes	Yes	Yes	Include	• Not aware of the researchers cultural and theoretical orientation.<br>• Researcher interpretation is not acknowledged. No reflection of role or response to events throughout study.
Tehrani (2015)	No	Yes	Yes	Yes	Yes	No	No	Yes	Yes	Yes	Include	• There is no philosophical orientation on which the study is based, just states qualitative methodology, can be considered less rigorous in design.<br>• Not aware of the researchers cultural and theoretical orientation.<br>• Researcher interpretation is not acknowledged. No reflection of role or response to events throughout study.
Wilkes (2010)	Yes	Yes	Yes	Yes	Yes	No	No	Yes	Yes	Yes	Include	• Not aware of the researchers cultural and theoretical orientation.<br>• Researcher interpretation is not acknowledged. No reflection of role or response to events throughout study.
