# Supplementary material for: Exploration of fathers’ mental health and well-being concerns during the transition to fatherhood, and paternal perinatal support: scoping review
Source: BMJ Open. 2024 Nov 12;14(11):e078386. doi: 10.1136/bmjopen-2023-078386 (PMC11574476; doi:10.1136/bmjopen-2023-078386)
Supplement: online supplemental file 3 [file bmjopen-14-11-s003.pdf]

Additional File 3. Table showing which studies are related to each theme and sub-theme.

First Author (Year)	Theme 1<br>1	Theme<br>2	Theme 2<br>1	Theme 2<br>2	Theme 3<br>1	Theme 3<br>2	Theme 4<br>1	Theme 4<br>2
Baldwin (2019) [4]	✓		✓	✓	✓	✓	✓	✓
Baldwin et al. (2021) [28]			✓		✓	✓	✓	✓
Baral (2021) [29]	✓		✓	✓			✓	
Barclay (1996) [30]	✓		✓		✓	✓	✓	
Clifford-Motop (2022) [58]	✓	✓	✓	✓	✓	✓	✓	✓
Dallos (2011) [31]	✓	✓	✓	✓			✓	
Darwin (2017) [10]	✓	✓	✓	✓	✓	✓	✓	✓
Davenport (2023) [59]	✓		✓	✓			✓	
Deave (2008) [32]	✓		✓	✓	✓	✓	✓	✓
Edhborg (2016) [19]	✓	✓	✓	✓		✓		
Fagerskiold (2008) [33]	✓	✓	✓			✓		✓
Fei-Wan a (2019) [34]	✓		✓	✓	✓			✓
Fenwick (2012) [35]		✓	✓		✓	✓	✓	
Finnbogadottir (2002) [36]	✓	✓	✓		✓	✓	✓	✓
Fletcher, (2019) [37]	✓	✓	✓					✓
Gottfredsdottir (2005) [38]	✓	✓	✓	✓	✓	✓		
Hall (1994) [39]	✓	✓	✓	✓				✓
Hodgson (2021) [40]		✓			✓	✓	✓	✓
Johannsson (2016) [41]		✓	✓					✓
Johansson (2020) [42]	✓		✓		✓		✓	
Kaner (2023) [60]	✓					✓	✓	✓
Kowlessar (2014) [43]		✓				✓		
Lagarto (2021) [44]		✓		✓	✓		✓	✓
Ling (2021) [45]	✓		✓	✓	✓		✓	✓
Machin (2015) [46]		✓	✓		✓		✓	✓
Nesporova (2019) [47]			✓				✓	
Pallson (2017) [48]	✓	✓	✓			✓	✓	
Pedersen (2021) [49]		✓	✓	✓	✓	✓	✓	
Rayburn (2021) [50]						✓	✓	✓
Reay (2023) [61]	✓				✓		✓	✓
Rominov (2018) [51]		✓		✓	✓	✓	✓	✓
Shorey (2017) [52]	✓		✓	✓	✓	✓	✓	✓
Shorey (2018) [53]		✓	✓	✓		✓		
Shorey (2019) [54]	✓	✓	✓	✓		✓		
St. John (2005) [55]	✓	✓	✓	✓			✓	✓
Tehrani (2015) [56]			✓					
Wilkes (2010) [57]	✓		✓	✓		✓	✓	

Around 2/3 of studies related
to each sub-theme
